# Dual Hypocretin Receptor Antagonism Is More Effective for Sleep Promotion than Antagonism of Either Receptor Alone

**DOI:** 10.1371/journal.pone.0039131

**Published:** 2012-07-02

**Authors:** Stephen R. Morairty, Florent G. Revel, Pari Malherbe, Jean-Luc Moreau, Daniel Valladao, Joseph G. Wettstein, Thomas S. Kilduff, Edilio Borroni

**Affiliations:** 1 Center for Neuroscience and Metabolic Disease Research, SRI International, Menlo Park, California, United States of America; 2 Neuroscience Research, F. Hoffmann-La Roche Ltd., Basel, Switzerland; Medical School of Hannover, United States of America

## Abstract

The hypocretin (orexin) system is involved in sleep/wake regulation, and antagonists of both hypocretin receptor type 1 (HCRTR1) and/or HCRTR2 are considered to be potential hypnotic medications. It is currently unclear whether blockade of either or both receptors is more effective for promoting sleep with minimal side effects. Accordingly, we compared the properties of selective HCRTR1 (SB-408124 and SB-334867) and HCRTR2 (EMPA) antagonists with that of the dual HCRTR1/R2 antagonist almorexant in the rat. All 4 antagonists bound to their respective receptors with high affinity and selectivity *in vitro.* Since *in vivo* pharmacokinetic experiments revealed poor brain penetration for SB-408124, SB-334867 was selected for subsequent *in vivo* studies. When injected in the mid-active phase, SB-334867 produced small increases in rapid-eye-movement (REM) and non-REM (NR) sleep. EMPA produced a significant increase in NR only at the highest dose studied. In contrast, almorexant decreased NR latency and increased both NR and REM proportionally throughout the subsequent 6 h without rebound wakefulness. The increased NR was due to a greater number of NR bouts; NR bout duration was unchanged. At the highest dose tested (100 mg/kg), almorexant fragmented sleep architecture by increasing the number of waking and REM bouts. No evidence of cataplexy was observed. HCRTR1 occupancy by almorexant declined 4–6 h post-administration while HCRTR2 occupancy was still elevated after 12 h, revealing a complex relationship between occupancy of HCRT receptors and sleep promotion. We conclude that dual HCRTR1/R2 blockade is more effective in promoting sleep than blockade of either HCRTR alone. In contrast to GABA receptor agonists which induce sleep by generalized inhibition, HCRTR antagonists seem to facilitate sleep by reducing waking “drive”.

## Introduction

Determination of the functions of neurotransmitters, neuromodulators and their receptors has classically been aided by use of small molecule receptor-specific antagonists. In recent years, forward and reverse genetics have provided insights into the functions of neurotransmitter/neuromodulatory systems before receptor-specific antagonists were developed. Such was the case for hypocretin (orexin), whose cell bodies in the perifornical and lateral hypothalamus synthesize a pair of neuropeptides alternatively called hypocretin-1 (HCRT1) or orexin-A and hypocretin-2 (HCRT2) or orexin-B [1], [2]. Identification of a mutation in the gene encoding HCRT receptor 2 (HCRTR2 or OX2R) as the cause of canine narcolepsy [3] and demonstration that HCRT ligand-deficient mice exhibited periods of behavioral arrest that resembled both human and canine narcolepsy [4] implicated the HCRT system in sleep/wake control well before the first small molecule HCRT receptor antagonists [5], [6], [7] were described. An extensive literature has since led to the conclusion that the HCRT system is wake-promoting [8], [9], and involved in energy homeostasis [12], [13]. Other studies have suggested roles for the HCRT system in neuroendocrine, cardiovascular, water balance, and gastrointestinal control [14], nociception and hyperalgesia [15], [16], [17], stress and stress-induced analgesia [18], [19], reward and addiction [20], [21], [22], [23], and panic anxiety [24].

It is currently unclear whether targeting the HCRTR2 alone or both HCRT receptors is the best strategy for the development of sleep-promoting compounds. Several dual HCRTR1/R2 antagonists show significant sleep-promoting properties [25], [26], [27], [28], [29], [30], [31], [32]. However, some reports indicate that HCRTR2 blockade alone was sufficient to produce the hypnotic actions of HCRTR antagonism [32], [33]. One study compared the efficacy of the selective HCRTR1 antagonist SB-408124 [34], the selective HCRTR2 antagonist JNJ-10397049 [35], and the dual antagonist almorexant [27] and concluded that HCRTR1 antagonism attenuates the hypnotic actions of HCRTR2 blockade [32]. While data on the affinity and selectivity of these compounds have been published, the absence of information on their pharmacokinetic properties is problematic for interpretation of their *in vivo* effects.

In the present study, we characterize the hypnotic activity of HCRTR antagonists in rats to determine whether selective or dual HCRTR antagonists are more effective for promoting sleep. To ensure a meaningful *in vivo* comparison, we determined the pharmacological and pharmacokinetic profiles in rats of two selective HCRTR1 antagonists, SB-408124 and SB-334867 [36], the selective HCRTR2 antagonist EMPA [37], and the dual HCRTR1/R2 antagonist almorexant. After showing that SB-408124 displays insufficient brain penetration, we used SB-334867 as the HCRTR1 antagonist for all *in vivo* experiments. Lastly, we determined the time course of HCRTR occupancy by almorexant and correlated this with hypnotic efficacy.

## Materials and Methods

### Drugs

Almorexant (ACT-078573, (2*R*)-2-[(1*S*)-6,7-Dimethoxy-1-[2-(4-trifluoromethyl-phenyl)-ethyl]-3,4-dihydro-1H-isoquinolin-2-yl]-*N*-methyl-2-phenyl-acetamide) [27], EMPA *N*-(Ethyl-2-[(6-methoxy-pyridin-3-yl)-(toluene-2-sulfonyl)-amino]-*N*-pyridin-3-ylmethyl-acetamide) [37], SB-674042 (1-(5-(2-fluoro-phenyl)-2-methyl-thiazol-4-yl)-1-((S)-2-(5-phenyl-(1,3,4)oxadiazol-2-ylmethyl)-pyrrolidin-1-yl)-methanone) [34], and Cp-5 ((*S*)-1-(6,7-Dimethoxy-3,4-dihydro-1H-isoquinolin-2-yl)-3,3-dimethyl-2-[(pyridin-4-ylmethyl)-amino]-butan-1-one) [7] were synthesized at F. Hoffmann-La Roche Ltd. (Basel, Switzerland) or SRI International (Menlo Park, CA USA) according to the patent literature [38]. SB-334867 (1-(2-methylbenzoxazol-6-yl)-3-[1], [5]naphthyridin-4-yl-urea hydrochloride), zolpidem (N,N,6-Trimehtyl-2-(methylphenyl)-imidazol[1,2-a]pyridine-3-acetamide) and SB-408124 (1-(6,8-difluoro-2-methyl-quinolin-4-yl)-3-(4-dimethylamino-phenyl)-urea) were purchased from Tocris Bioscience (Ellisville, MO). Chemical structures are provided in Figure S1. [^3^H]almorexant (specific activity: 42.7 Ci/mmol), [^3^H]SB-674042 (specific activity: 24.4 Ci/mmol) and [^3^H]EMPA (specific activity: 94.3 Ci/mmol) were synthesized at Roche.

### Animals

Animal experiments performed at F. Hoffmann-La Roche were conducted in strict adherence to the Swiss federal regulations on animal protection and to the rules of the Association for Assessment and Accreditation of Laboratory Animal Care International (AAALAC), and with the explicit approval of the local Cantonal Veterinary Office/Authority Basel City. Animal experiments performed at SRI International were approved by SRI’s Institutional Animal Care and Use Committee and were in accordance with U.S. National Institute of Health guidelines. Male Wistar rats (240±20 g) used for spontaneous locomotion studies and pharmacokinetic studies at F. Hoffmann-La Roche were obtained from RCC Ltd. (Fullinsdorf, Switzerland). Male Sprague-Dawley rats (300±25 g) used for receptor occupancy studies at F. Hoffmann-La Roche were from Iffa Credo (Lyon, France). Animals were housed in separate rooms under a 12 h light/12 h dark cycle (light onset: 06∶00, except where noted below; Zeitgeber time 0, ZT0) at 22±2°C, with *ad libitum* access to food and water. Male Sprague-Dawley rats (300±25 g) used for sleep studies at SRI were from Charles River (Wilmington, MA) and were housed in a temperature-controlled recording room under a 12 h light/12 h dark cycle (lights on at 05∶00) with food and water available *ad libitum*. Room temperature (24±2°C), humidity (50±20% relative humidity), and lighting conditions were monitored continuously via computer. Animals were inspected daily in accordance with AAALAC and SRI guidelines.

### Pharmacological Studies

**[^3^H]almorexant binding to rat HCRTR1 and HCRTR2.** The rat cDNAs encoding HCRTR1 (Accession No. P56718) and HCRTR2 (Accession No. P56719) were subcloned into pCI-Neo expression vectors (Promega, Madison, WI) and used to transfect HEK293 cells (acquired commercially from ATCC-LGC, Molsheim, France) as previously described [37]. Membrane preparations, saturation and inhibition experiments, and determination of the association and dissociation kinetic parameters of [^3^H]almorexant to rHCRTR2-HEK293 cell membranes were performed at F. Hoffmann-La Roche as previously described [37] and reported in the Materials and Methods S1.

### Pharmacokinetic Studies

Pharmacokinetic analyses were performed at F. Hoffmann-La Roche as described in supporting Materials and Methods S1.

#### SB-334867 selectivity screen

SB-334867 was evaluated in a selectivity screen performed at CEREP (Paris, France). The screen consisted of binding assays on a panel of 79 target receptors. The specific binding (SB) of a radioligand to each target receptor was defined as the difference between the total binding and the nonspecific binding determined in the presence of a cold competitor in excess. The results are expressed as a percent of control SB obtained in the presence of SB-334867 used at 10 µM. Details on the CEREP screen are available from www.cerep.fr.

### Effect of Almorexant and SB-334867 on Spontaneous Locomotor Activity in Rats

Locomotor activity (LMA) was evaluated at F. Hoffmann-La Roche as described previously [39]. Male Wistar rats were placed for 2 weeks in a 12 h light/12 h dark cycle with light onset at 22∶00 (ZT0). Three h after the onset of the dark period (i.e., ZT15), rats were injected ip with either vehicle or HCRT receptor antagonist (almorexant or SB-334867 at 3, 10, 30 mg/kg in NaCl 0.9%, 0.3% Tween-80) (n = 8 per group), and returned to the recording chambers. Spontaneous LMA was recorded for a period of 30 min. At the end of the experiment, the brain and plasma were collected for determination of the drug exposure and brain/plasma concentration ratio.

### Electroencephalogram, Core Body Temperature and Locomotor Activity Studies

#### Surgical procedures and recordings

All rodent electroencephalograph (EEG) studies were performed at SRI International. Three groups of eight male Sprague-Dawley rats (300±25 g; Charles River, Wilmington, MA) were implanted with chronic recording devices (F40-EET, Data Sciences Inc., St Paul, MN) for continuous recordings of EEG, electromyograph (EMG), core body temperature (T_core_), and LMA via telemetry as previously described [40]. Data recording and scoring were performed as previously reported [40] (see also Supplemental Material and Methods). The EEG and EMG data were scored in 10 sec epochs for waking (W), rapid eye movement sleep (REM), and non-REM sleep (NR). T_core_ and LMA (counts per minute) were analyzed as hourly means. Data from the EEG studies are reported in hourly means such that the hourly time ZT1 refers to the hour between time points ZT0 and ZT1.

#### Experimental design

For each of the three separate studies, a repeated measures counter-balanced design was employed in which each rat received five separate dosings. The dosing conditions for study 1 included SB-334867 at three concentrations (3, 10 and 30 mg/kg), zolpidem (ZOL, 7.5 mg/kg) and a vehicle control (saline 95%/ethanol 5%). The dosing conditions for study 2 included EMPA at three concentrations (10, 30 and100 mg/kg), ZOL (10 mg/kg) and a vehicle control (HPMC). The dosing conditions for study 3 included almorexant at three concentrations (10, 30 and 100 mg/kg), ZOL (10 mg/kg) and a vehicle control (HPMC). All dosings were administered ip in a volume of 2 ml/kg. A minimum of 3 d elapsed between doses. Dosing occurred during the middle of the rats’ normal active period at the start of ZT19 and was typically completed within 10 min. Animals were continuously recorded for 6 h prior to dosing and for 18 h following dosing.

#### Determination of HCRTR1 and HCRTR2 occupancy by almorexant

This study was performed at F. Hoffmann-La Roche. Sixty-five male Sprague-Dawley rats, housed 5 per cage (light onset: 12∶00), were injected intraperitoneally (ip) with either vehicle (1.25% hydroxypropyl methylcellulose (HPMC), 0.1% docusate sodium) or almorexant (30 mg/kg in 1.25% HPMC, 0.1% docusate sodium) at the mid-dark phase (ZT18; i.e., 6 h after lights-off), and returned to their home cage. Groups (n = 5 per group) of vehicle- or almorexant-treated animals were then sacrificed by decapitation 0.5, 2, 4, 8 or 12 h after the injection. An extra group of non-injected rats (n = 5) was also sampled at ZT18. Plasma was collected and stored at −80°C until assayed. Brains were rapidly dissected, frozen on dry ice, and stored at −80°C. Series of coronal brain sections (14 µm) were cut in a cryostat through the posterior hypothalamus (tuberomammillary nucleus level: 3.8 to 4.2 mm posterior to bregma) and the brain stem (dorsal raphe nucleus level: 7.3 to 8 mm posterior to bregma; locus coeruleus level: −9.3 to −10 mm posterior to bregma), thaw-mounted (6 sections per slide) and stored at −20°C. After sectioning, the remaining pieces of brain were kept at −80°C for later determination of almorexant brain concentration. The brain and plasma concentrations of almorexant were determined by quantitative liquid chromatography/mass spectrometry/mass spectrometry (LC/MS/MS).

Receptor occupancy (RO) was determined as published previously [41]. For each Hcrt receptor subtype, two series of slides were thawed and incubated at room temperature with the relevant radioligand in assay buffer for 15 min (HCRTR1) or 1 h (HCRTR2). For HCRTR1, assay buffer (2****mM CaCl_2_, 5 mM MgCl_2_, 25 mM HEPES, pH 7.4, 100 µL per section) contained either 5 nM [^3^H]SB-674042 (for determination of total binding, TB) or 5 nM [^3^H]SB-674042 plus 10 µM SB-408124 (for determination of non-specific binding, NSB). For HCRTR2, assay buffer (1 mM CaCl_2_, 5 mM MgCl_2_, 25 mM HEPES, pH 7.4, 120 µL per section) contained either 1 nM [^3^H]EMPA (for determination of TB) or 1 nM [^3^H]EMPA plus 10 µM Cp-5 (for determination of NSB). The liquid was drained, the brain sections were rinsed with ice-cold assay buffer (2 brief washes followed by 3×2 min soaking) and distilled water (3 brief dips) and air dried at 4°C for 12 h. The slides were exposed together with [^3^H] microscales against tritium-sensitive imaging plates (BAS-TR2025) for 5 days. The plates were scanned with a high resolution phosphor imager device (Fujifilm BAS-5000) and calibrated measurements of radioactivity (fmol/mg protein) were made. All analyses were performed blind to treatment.

For each selected region, the mean signal density (TB) was measured and averaged from three consecutive sections from the same slide. The specific binding (SB) signal was then determined for each animal by subtracting the NSB signal from the TB signal. NSB was measured from adjacent brains sections incubated with the radiotracer and an excess of cold competitor. The SB signal was averaged for each experimental group and the percent RO was calculated at each time-point according to the equation RO = (1-(SB_almorexant_/SB_vehicle_))×100, where SB_almorexant_ is the average SB for the animal group injected with almorexant and SB_vehicle_ is the average SB for the animal group injected with vehicle.

### Statistical Analyses

Results are shown as mean±SEM. LMA and RO data were analyzed with one-way ANOVA followed by Dunnett’s analysis. EEG data were analyzed with repeated measures (rm)-ANOVA, followed by paired two-tailed *t*-tests. REM:NR ratios, sleep latencies (NR and REM) and cumulative data were analyzed with one-way rm-ANOVA and all other data with two-way rm-ANOVA. Light period and dark period data were analyzed separately as well as pre- and post-drug administration data. Statistical significance was set at *P*<0.05.

## Results

### Pharmacological Studies

#### Binding characteristics of [^3^H]almorexant to rHCRTR1- and rHCRTR2-expressing cell membranes

**Figure 1:**
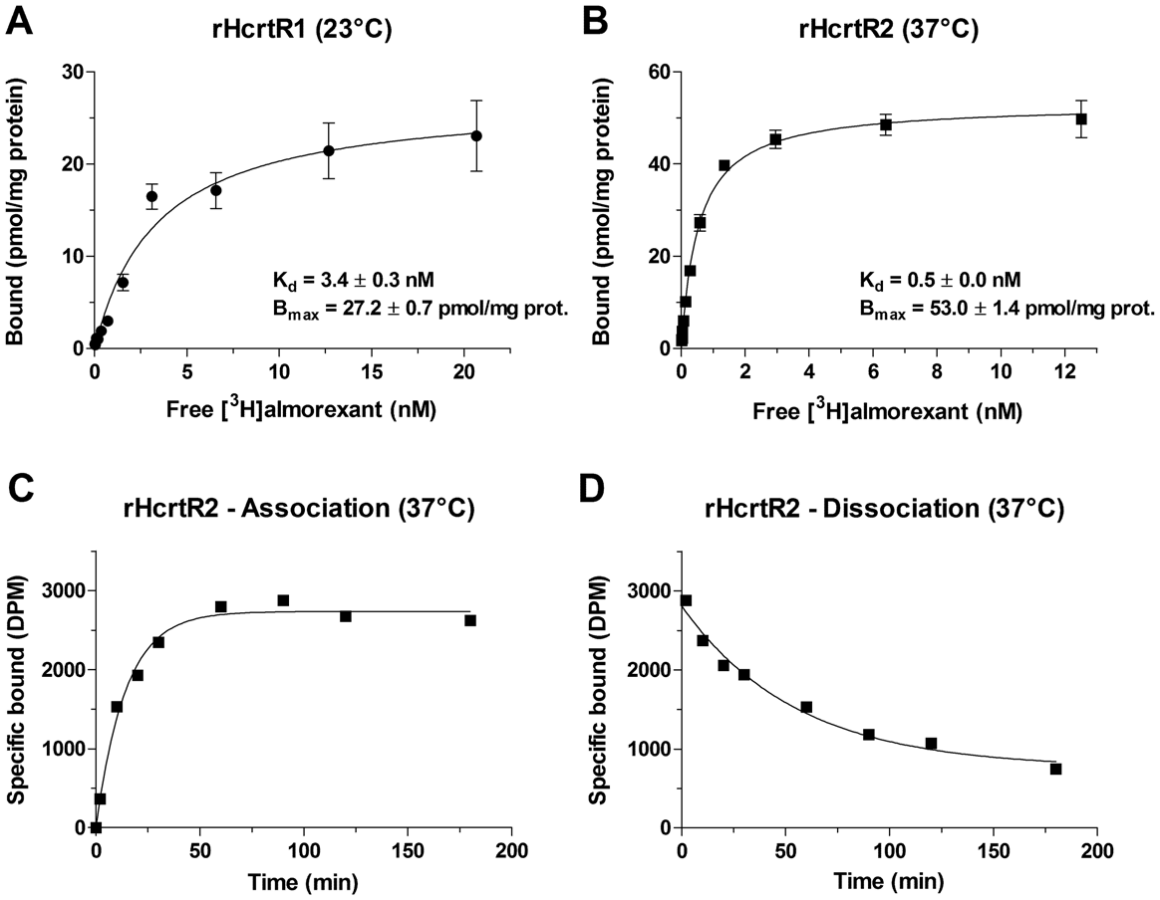
Binding characteristics of [^3^H]almorexant to rHCRTR1 and rHCRTR2 cell membranes. (**A,B**) Saturation binding curves of [^3^H]almorexant binding to membranes from HEK293 cells transiently transfected with rHCRTR1 (**A**) or rHCRTR2 (**B**). Each data point represents the mean±SEM of three independent experiments performed in triplicate. The data were analyzed by nonlinear regression analysis using GraphPad Prism 4.0 software and a single-site binding model. (**C,D**) Time course for the association (**C**) and dissociation (**D**) of [^3^H]almorexant binding to rHCRTR2 membranes.

To characterize the *in vitro* binding of [^3^H]almorexant to rat HCRT receptors, saturation binding analyses were performed at binding equilibrium on membranes isolated from HEK293 cells transiently transfected with rHCRTR1 and rHCRTR2. As shown in Fig. 1A and B, [^3^H]almorexant bound with high affinity to a single saturable site on recombinant rHCRTR1 (K_d_ of 3.4±0.3 nM and B_max_ of 27.2±0.7 pmol/mg prot, at 23°C) and rHCRTR2 (K_d_ of 0.5±0.0 nM and B_max_ of 53.0±1.4 pmol/mg prot, measured at 37°C). Binding kinetics of [^3^H]almorexant to membrane preparations from HEK293 cells transiently expressing rHCRTR2 are shown in Fig. 1C and D and the kinetic parameters in Table 1. The association binding of [^3^H]almorexant to the rHCRTR2 had a half-maximal binding at 10 min and reached equilibrium within 50 min. The data were fit to a one-phase exponential model with the association rate constant of 0.073±0.015 nM^−1^min^−1^. The dissociation rate for [^3^H]almorexant binding to the rHCRTR2 was determined by the addition of an excess amount of almorexant (5 µM) after equilibrium was reached. The rate of [^3^H]almorexant dissociation from rHCRTR2 membrane was slow; the reversal of binding was incomplete and did not reach baseline even after 2 h (Fig. 1D & Table 1).

**Table 1:** Kinetic parameters for the association and dissociation of [^3^H]almorexant in rHCRTR2-HEK293 cell membranes at 37°C.

Compound	Association kinetic	Dissociation kinetic	Apparent	
	K_on_ (nM^−1^min^−1^)	K_off_ (min^−1^)	t_1/2_ (min)	K_d_ (nM)
**[^3^H]almorexant**	0.073±0.015	0.021±0.004	36.3±5.7	0.33±0.9

The K_on_ (calculated on rate), K_off_ (observed off rate), t_1/2_ (half-maximal binding) and K_d_ (apparent dissociation constant) values are ± SEM, calculated from three independent experiments (each performed in quadruplicate) as described under “Materials and Methods”.

The potencies of almorexant and of the selective HCRTR1 antagonists SB-334867 [6] and SB-408124 [34] in inhibiting [^3^H]almorexant binding to HEK293-rHCRTR1 and HEK293-rHCRTR2 cell membranes are given in Table 2. Almorexant was able to displace [^3^H]almorexant binding from rHCRTR1 and rHCRTR2 membranes with high affinity (Table 2). In contrast, SB-334867 and SB-408124 displaced [^3^H]almorexant binding from rHCRTR1, but not from rHCRTR2, with high affinity (Table 2).

**Table 2:** Potencies of almorexant, SB-408124 and SB-334867 antagonists in inhibition of [^3^H]almorexant binding to the membrane preparations from HEK293 cells transiently expressing rHCRTR1 and rHCRTR2.

Compound	rHCRTR1	rHCRTR2
	[^3^H]almorexant (23°C)	[^3^H]almorexant (37°C)
	K_i_ (nM)	K_i_ (nM)
**almorexant**	7.1±0.7	2.0±0.0
**SB-408124**	45.7±4.1	5370.0±2200.0
**SB-334867**	58.4±2.9	2390.0±81.0

[^3^H]almorexant was used at a concentration equal to its K_d_ values of 3.4 nM and 0.5 nM at rHcrtR1 and rHcrtR2, respectively, in these competition binding experiments. K_i_ values for [^3^H]almorexant binding inhibition by various antagonists were calculated as described under “Materials and Methods”. Values are ± SEM of the K_i_ calculated from three independent experiments, each performed in duplicate.

### Pharmacokinetic Studies

#### Pharmacokinetic properties of SB-334867, SB-408124, EMPA and almorexant in rats

The oral bioavailability and pharmacokinetic properties of almorexant, SB-334867 and SB-408124 were evaluated in Wistar rats. The mean pharmacokinetic parameters after single iv or oral (po) bolus administration in rat are given in Table S1. Almorexant displayed a high systemic plasma clearance, high volume of distribution at steady state (Vss) and low oral bioavailability in rat. In addition, almorexant was highly bound to plasma proteins (<3.7%, and <8.7% free fraction in human and rat plasma, respectively), and its stability measured for 2 h in human and rat plasma was 90.0% and 95.0%, respectively. The mean brain/plasma concentration ratio of almorexant was 0.12 in rat.

SB-334867 exhibited a low systemic plasma clearance, medium Vss and oral bioavailability in rat. SB-334867 is highly bound to plasma proteins (1.3%, and 0.8% free fraction in human and rat plasma, respectively), and its stability measured for 1 h/4 h in human and rat plasma was 95%/93% and 104%/110%, respectively. The mean brain/plasma concentration ratio of SB-334867 (at a dose of 8.8 mg/kg, po) was 0.53 in rat.

SB-408124 had a low systemic plasma clearance, low Vss and medium oral bioavailability in rat. SB-408124 had very low free fraction in human and rat (0.3% and <0.1%, respectively) and its stability (1 h/4 h) in human and rat plasma was 94%/88% and 101%/107%, respectively. The mean brain/plasma concentration ratio of SB-408124 (at dose of 18 mg/kg, po) was 0.03 in rat. Such unfavorable pharmacokinetic properties of SB-408124, most importantly its extremely low brain penetration, prompted us to use SB-334867 for further *in vivo* studies in the rat.

The pharmacokinetic profiles of EMPA have been reported previously [37].

#### Selectivity profile of SB-334867

The specificity of SB-334867 at the HCRTR1 was confirmed by assessment in radioligand binding assays in a broad CEREP screen (Paris, France; www.cerep.fr) (Table S2). Among the 79 receptors tested, 30 were peptide receptors. SB-334867 was inactive (<40% activity at 10 µM) at all targets tested with the exception of the A_2A_ (adenosine), A_3_, MT3 (melatonin), P_2Y_ (purinergic 2Y) and 5HT_2C_ (serotonin 2C) receptors, where it caused 89%, 63%, 102%, 64% and 70% displacement of specific binding at 10 µM, respectively. The selectivity profiles of almorexant [27] and EMPA [37] have been reported previously.

### Effect of Almorexant and SB-334867 on Spontaneous Locomotor Activity in Rats

**Figure 2:**
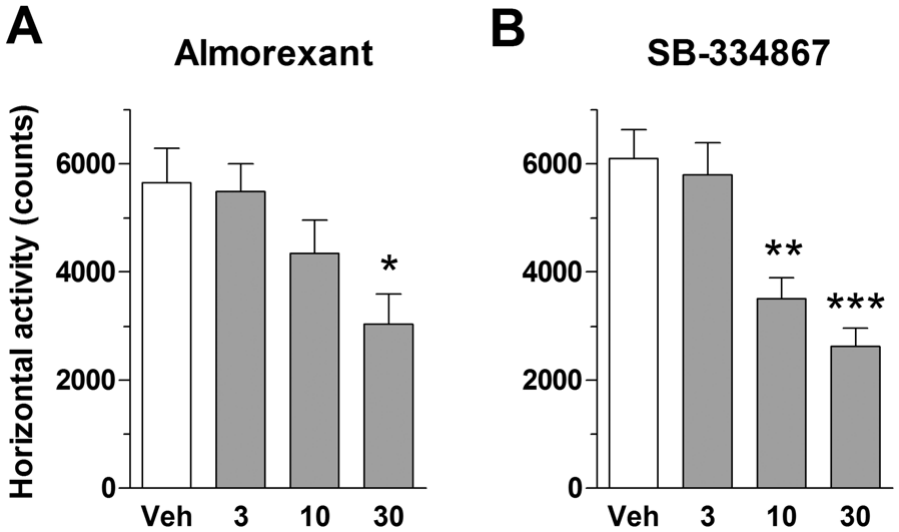
Effects of almorexant and SB-334867 on spontaneous locomotor activity of rats during the active phase. Both almorexant **(A)** and SB-334867 **(B)** reduced locomotor activity compared to vehicle (Veh) when administered 3 h after the onset of the dark period. Horizontal locomotor activity was recorded for a period of 30 min. Numbers on the X-axes represent intraperitoneal doses in mg/kg.***p<0.001, **p<0.01, *p<0.05 vs. Veh (one-way ANOVA followed by Dunnett’s analysis). All data are mean±SEM (n = 8 per group).

The ability of almorexant and SB-334867 to antagonize *in vivo* the biological action of endogenous hypocretins was assessed by measuring spontaneous LMA during the active phase. Almorexant dose-dependently reduced LMA, although only the 30 mg/kg dose reached significance when compared to vehicle (Figure 2A; F = 4.28, p<0.05). Similarly, SB-334867 dose-dependently reduced spontaneous LMA, with both the 10 and 30 mg/kg doses being statistically different from vehicle (Figure 2B; vehicle: 6097±536; 10 mg/kg: 3509±383; 30 mg/kg: 2626±341; F = 12.80, p<0.01 and p<0.001, respectively).

The plasma and brain exposure of SB-334867 were measured at the end of the LMA experiment. When determined 35 min after ip administration, SB-334867 doses of 3, 10 and 30 mg/kg produced plasma levels of 220, 718 and 738 ng/mL vs. brain levels of 48, 171, and 142 ng/mL (ratios: 0.21, 0.23, 0.19, respectively). These results confirmed the ability of SB-334867 to enter the rat brain at the doses used in this report.

### Rodent EEG Studies

**Figure 3:**
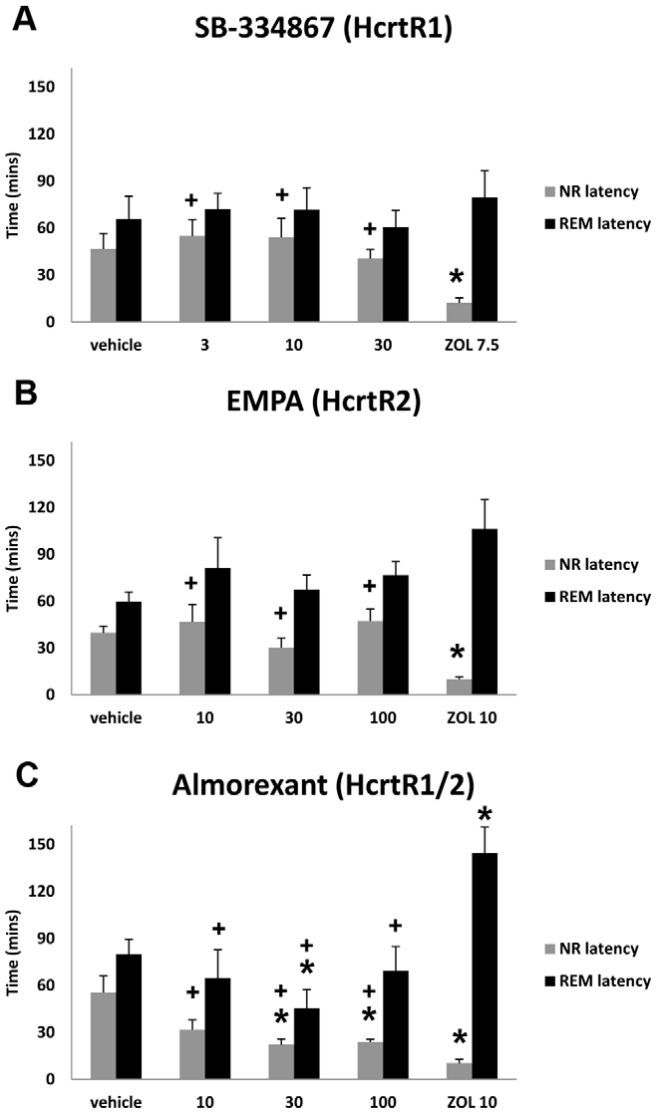
Latency to the onset of NR and REM sleep following administration of SB-334867. (A) , EMPA **(B)**, and almorexant **(C)** as compared to zolpidem (ZOL). * = significantly different from vehicle (p<0.05); **+** = significantly different from ZOL (p<0.05) (One-way repeated measures ANOVA followed by paired two-tail *t* tests; n = 8 per group). Data represent the mean±SEM.

The effects of almorexant, SB-334867 and EMPA administered in the middle of the dark (active) period were evaluated during the latter half of the active period and subsequent light (inactive) period to determine both efficacy for sleep promotion and whether “hangover” or rebound effects occurred. Of these three compounds, only almorexant reduced NR and REM sleep latency (Figure 3). Almorexant at 30 and 100 mg/kg reduced NR latency while only the 30 mg/kg concentration decreased latency to REM sleep. ZOL produced a decrease in NR latency in all three experiments.

**Figure 4:**
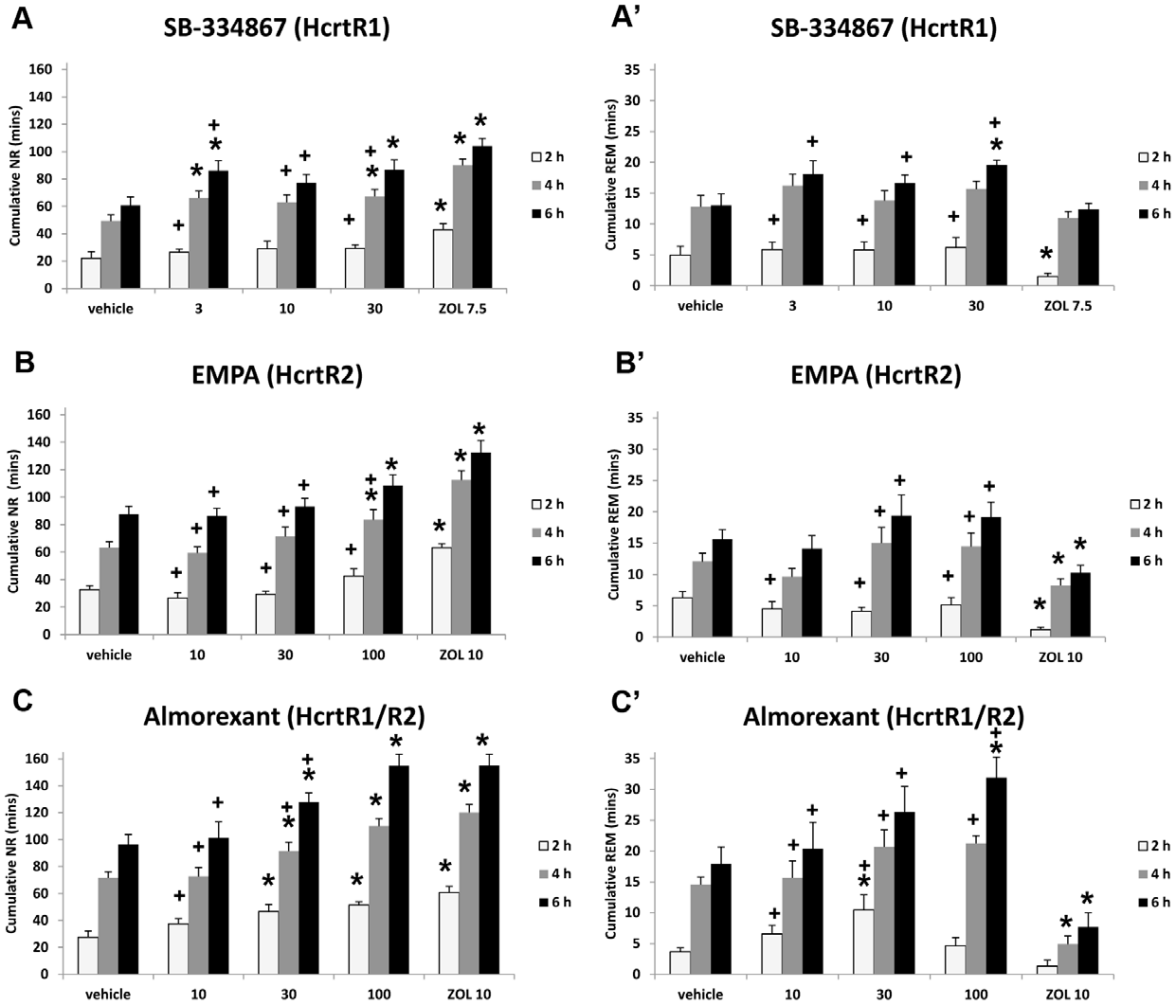
Cumulative time in NR and REM sleep over the first 2, 4 and 6 h following drug administration. **(A–C)** Cumulative time spent in NR sleep following SB-334867 (**A**), EMPA (**B**) and almorexant **(C)** compared to zolpidem (ZOL). **(A’–C’)** Cumulative time spent in REM sleep for the same drug treatments. (One-way repeated measures ANOVA followed by paired two-tail *t* tests; n = 8 per group). Data represent the mean±SEM. *, significantly different from vehicle; **+**, significantly different from ZOL.

In contrast, all three compounds increased NR sleep (Figure 4). SB-334867 at 3 and 30 mg/kg increased cumulative NR for the first 4 and 6 h periods following administration (F = 10.808, p<0.0001 and F = 10.752, p<0.0001, respectively). EMPA at 100 mg/kg also increased cumulative NR for the first 4 and 6 h periods post administration (F = 17.655, p<0.0001 and F = 12.816, p<0.0001, respectively). Almorexant had the strongest effect: both 30 and 100 mg/kg increased cumulative NR for 2, 4 and 6 h following administration (F = 13.010, p<0.0001; F = 17.771, p<0.0001; and F = 16.179, p<0.0001, respectively). Cumulative REM also increased for the first 2 h following almorexant at 30 mg/kg (F = 5.418, p = 0.0023) and for the 6 h period following the 100 mg/kg dose (Figure 4; F = 8.535, p<0.0001). ZOL increased cumulative NR and decreased cumulative REM in all three experiments. Whereas ZOL suppressed the REM:NR ratio in all 3 studies, none of the 3 test compounds did (Table 3). Although ZOL had significant effects on EEG delta power during NR, this parameter was little affected by any of the three test compounds compared to vehicle control (Figure S2).

**Table 3:** REM:NR ratios for the 6 h period following the administration of SB-334867, EMPA and almorexant.

Vehicle	SB-334867	SB-334867	SB-334867	ZOL
	**3 mg/kg**	**10 mg/kg**	**30 mg/kg**	**7.5 mg/kg**
0.22±0.039	0.21±0.016**^+^**	0.22±0.023**^+^**	0.23±0.016**^+^**	0.12±0.009*
**Vehicle**	**EMPA**	**EMPA**	**EMPA**	**ZOL**
	**10 mg/kg**	**30 mg/kg**	**100 mg/kg**	**10 mg/kg**
0.18±0.018	0.16±0.019**^+^**	0.21±0.032**^+^**	0.18±0.027**^+^**	0.08±0.008*
**vehicle**	**Almorexant**	**Almorexant**	**Almorexant**	**ZOL**
	**10 mg/kg**	**30 mg/kg**	**100 mg/kg**	**10 mg/kg**
0.18±0.020	0.19±0.026**^+^**	0.20±0.022**^+^**	0.21±0.021**^+^**	0.05±0.013*

* = significantly different from vehicle (p<0.05), **^+^** = significantly different from ZOL (p<0.05).

**Figure 5:**
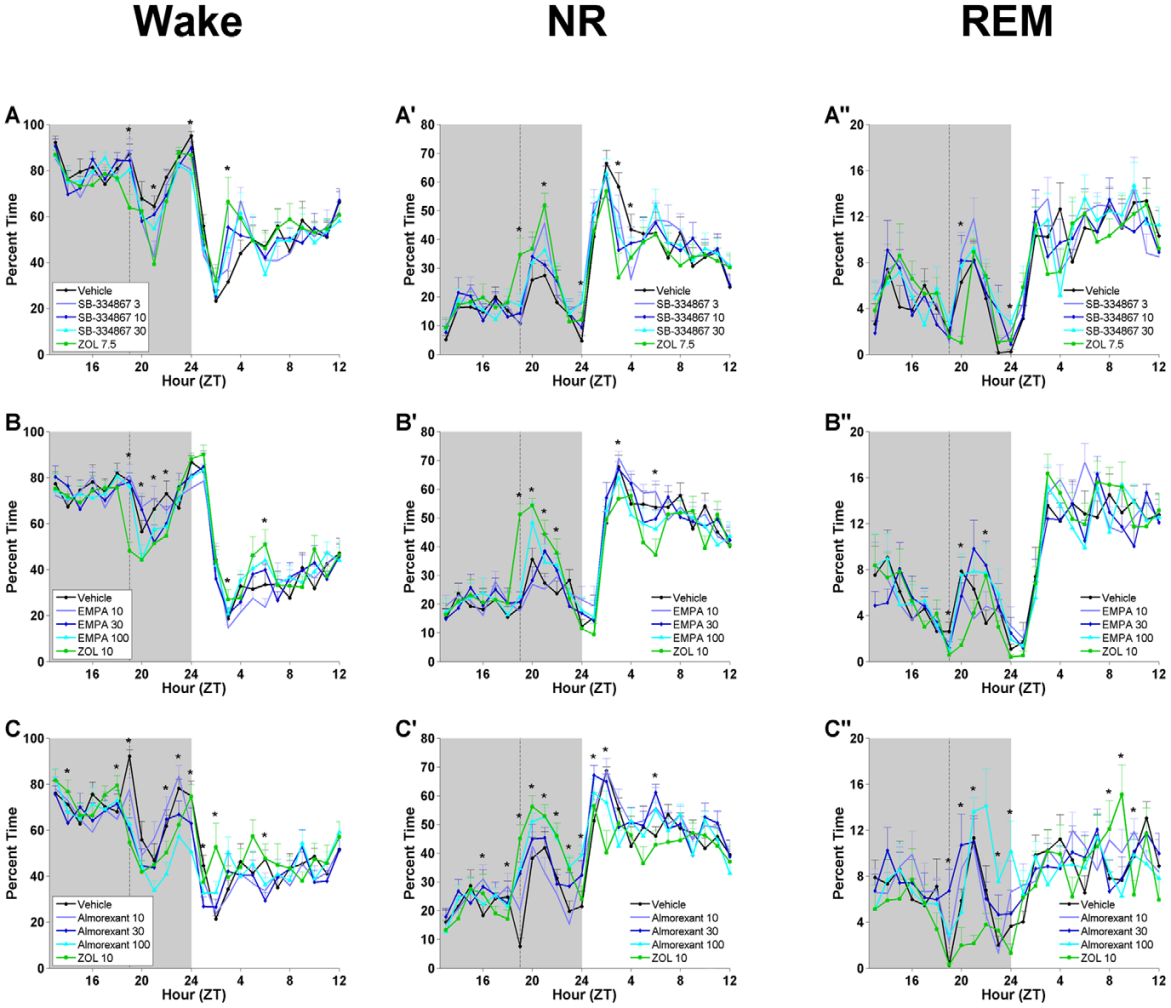
Hourly distribution of W, NR and REM sleep. W, NR and REM sleep for 6 h prior to and 18 h after administration of SB-334867 **(A)**, EMPA **(B)**, and almorexant **(C)** as compared to zolpidem (ZOL) and vehicle. Shaded area represents the dark phase; vertical dotted line in each panel indicates the time of injection. **(A)** Hourly amounts of wakefulness following SB 334867. **(A’)** Hourly amounts of NR sleep following SB 334867. **(A’’)** Hourly amounts of REM sleep following SB 334867. **(B)** Hourly amounts of wakefulness following EMPA. **(B’)** Hourly amounts of NR sleep following EMPA. **(B’’)** Hourly amounts of REM sleep following EMPA. **(C)** Hourly amounts of wakefulness following almorexant. **(C’)** Hourly amounts of NR sleep following almorexant. **(C’’)** Hourly amounts of REM sleep following almorexant. Data represent the mean±SEM (n = 8 rats per group). *, *p*<0.05. For detailed statistical results, see Text S1.

There were few effects on sleep/wake amounts during the light period subsequent to administration of EMPA, SB-334867 or almorexant (Figure 5). REM was not significantly affected during this period following any of the three HCRT antagonists. NR decreased during the third hour of the light period (ZT3) following SB-334867 at 10 and 30 mg/kg while NR increased during ZT1 and ZT6 following almorexant at 30 mg/kg compared to vehicle. No significant effects on NR were found following EMPA during the light period.

Significant results occurred in measures of sleep-wake consolidation (Tables S3, S4, S5 and Figures S3, S4, S5). The strongest effects were found following almorexant at 100 mg/kg, which produced increased numbers of W and NR bouts during ZT19, ZT20, and ZT22-ZT24 (F = 2.069, p = 0.0077 and F = 2.413, P = 0.0015, respectively). The number of REM bouts was increased by almorexant at 100 mg/kg during ZT22-ZT24 (F = 2.963, p = 0.002). W bout duration was decreased following almorexant at 100 mg/kg during ZT22 compared to vehicle (F = 2.320, p = 0.0023). All three concentrations of EMPA increased the number of W bouts (F = 4.243, p = 0.0065). SB-334867 increased NR bout duration during ZT21 following 30 mg/kg and during ZT24 following 3 mg/kg (F = 4.574, p<0.0001).

**Figure 6:**
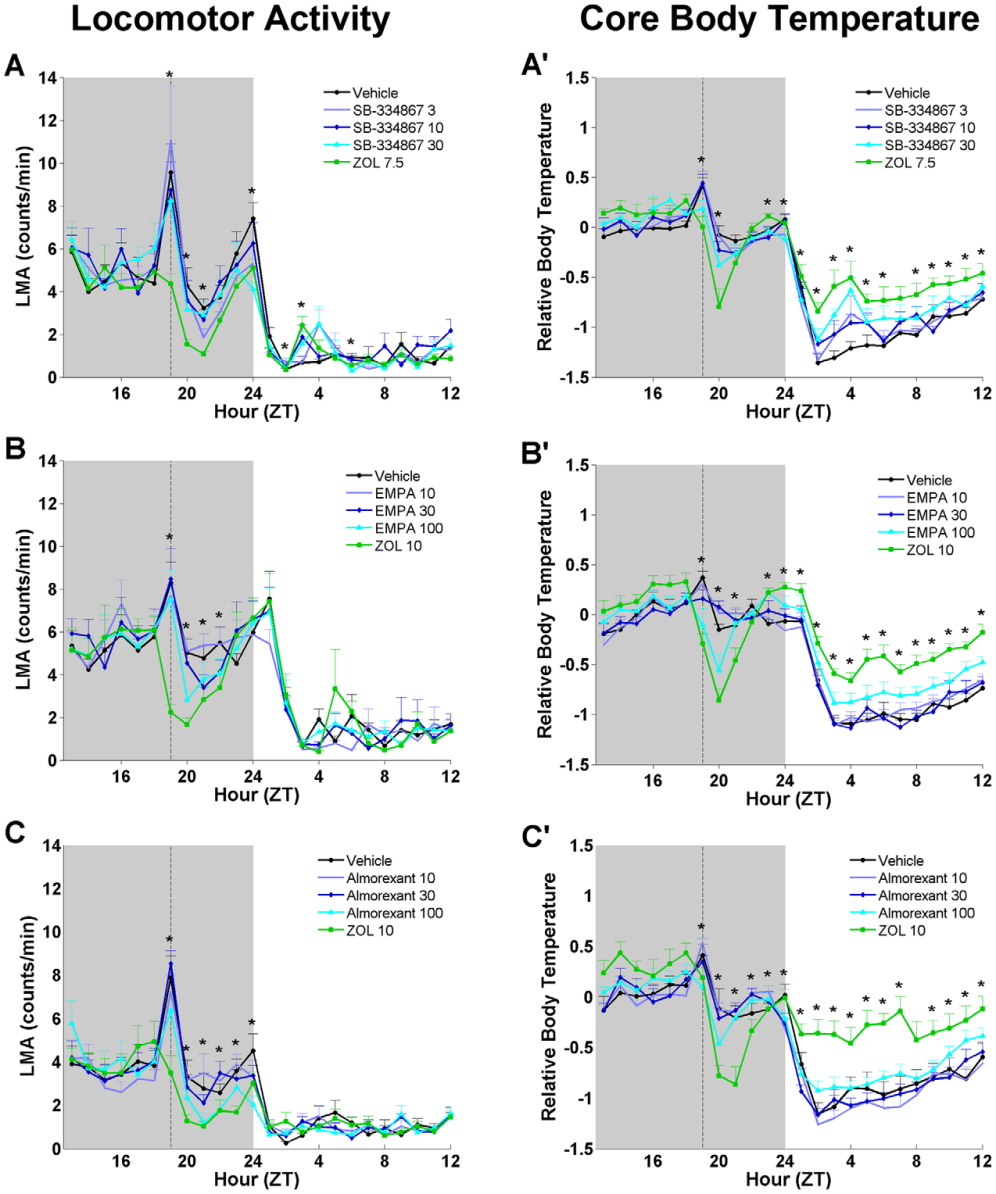
Average hourly LMA and relative T_core_. LMA and relative T_core_ for 6 h prior to and 18 h after administration of SB-334867 (A), EMPA (B), and almorexant (C) as compared to zolpidem (ZOL) and vehicle. Shaded area represents the dark phase; vertical dotted line in each panel indicates the time of injection. (A) Average hourly LMA following SB-334867. (A’) The average hourly T_core_ following SB-334867. (B) The average hourly LMA following EMPA. (B’) The average hourly T_core_ following EMPA. (C) The average hourly LMA following almorexant. (C’) The average hourly T_core_ following almorexant. Data represent the mean±SEM (n = 8 rats per group). *, *p*<0.05. For detailed statistical results see Text S1.

Both LMA and T_core_ underwent dose-dependent decreases after drug treatment (Figure 6). ANOVA revealed condition effects for both almorexant and EMPA in which LMA was decreased across the 6 h period following administration of both compounds at 100 mg/kg compared to vehicle (F = 7.316, p<0.00015 and F = 7.442, p = 0.00018 respectively). No differences in LMA during the subsequent light period were found. Condition effects for T_core_ were found in all three studies. The high concentrations tested for all three HCRT receptor antagonists decreased T_core_ across the 6 h period following administration (F = 7.629, p = 0.00027 for SB-334867; F = 7.442, p = 0.00018 for EMPA; F = 7.315, p = 0.00036 for almorexant). ZOL administration resulted in the largest declines in T_core_ in all three studies, which was followed by a sustained rebound increase in T_core_ during the subsequent light period.

### Time Course of HCRT Receptor Occupancy (RO) by Almorexant

**Figure 7:**
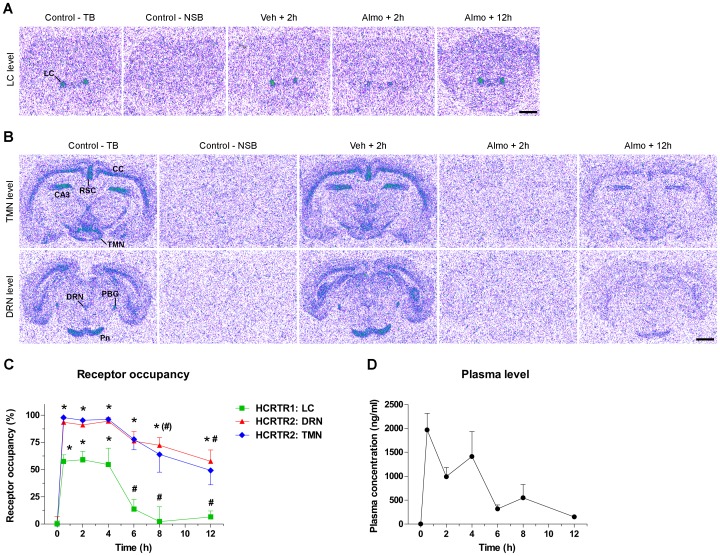
Time-course of HCRT1R and HCRT2R occupancies by almorexant. **(A,B)** Representative autoradiograms showing [3H]SB-674042 (5 nM) binding to HCRTR1 **(A)** and [3H]EMPA (1 nM) binding to HCRTR2 **(B)** in rat coronal brain sections. For both receptors, total binding (*TB*) was maximal in control animals (not injected) sampled at time 0 (*t0*). For HCRTR1 **(A)**, a clear signal was evident in the locus coeruleus (*LC*), which could be displaced by co-incubation with an excess of cold SB-674042 (10 µM) (non-specific binding, *NSB*). In contrast to vehicle administration (*Veh, 2 h*), almorexant (30 mg/kg injected intraperitoneally at ZT18) attenuated such specific signal after 2 h (*Almo, 2 h*), but not after 12 h (*Almo, 12 h*). For HCRTR2 **(B)**, signal was observed in various brain regions, including the tuberomammillary nuclei (*TMN*), cerebral cortex (*CC*), field CA3 of the hippocampus (*CA3*), retrosplenial cortex (*RSC*), dorsal raphe nuclei (*DRN*), pontine nuclei (*Pn*) and parabigeminal nuclei (*PBG*). [3H]EMPA could be displaced by co-incubation with an excess of Cp5 (10 µM) (*NSB*). HCRTR2 binding became minimal 2 h after almorexant (*Almo, 2 h*), but not after Vehicle (*Veh+2 h*), administration. After 12 h (*Almo, 12 h*), HCRTR2 binding was intermediate. Scale bars, 2 mm. **(C)** Time course of HCRTR1 and HCRTR2 occupancies by almorexant. Receptor occupancy was calculated by measuring the specific binding at various time points in the *LC* for HCRTR1, and in the *TMN* and *DRN* for HCRTR2. *, *p*<0.001 versus time 0; (#), *p*<0.05 (TMN only), #, *p*<0.05 (TMN) or *p*<0.01 (DRN), vs. time 30 min (one-way ANOVA followed by Dunnett’s analysis). **(D)** Almorexant plasma concentrations. Data represent the mean±SEM (n = 5 rats per group).

To determine the time-course of HCRTR1 and HCRTR2 RO by almorexant, a single dose of almorexant at the smallest concentration shown to promote sleep (30 mg/kg, ip; Figure 4) was administered in the mid-dark phase (ZT18) and rats were sacrificed after incubation periods of 0, 0.5, 2, 4, 8 or 12 h. For both HCRTR1 and HCRTR2, the NSB was minimal and represented 6.2% and 3%, respectively, of the average TB signal measured in control animals. The signal localization was in good agreement with the distribution of *HCRTR1*- and *HCRTR2*-expressing neurons [42], [43], as confirmed by *in situ* hybridization on separate sections (data not shown). Figure 7A shows representative autoradiograms of HCRTR1 binding sites in the locus coeruleus (*LC)*. This signal localization is in good agreement with the distribution of *Hcrtr1*-expressing neurons [42], [43], as confirmed by *in situ* hybridization (data not shown). The rats injected with vehicle displayed maximal HCRTR1 radiotracer binding at all time points (Figure 7A), whereas the animals injected with almorexant showed reduced binding 2 h after the injection. Binding of the HCRTR1 radiotracer returned to levels similar to control 8–12 h post almorexant injection.

Figure 7B shows representative autoradiograms of the HCRTR2 binding sites examined at 2 different rostro-caudal levels. At the level of the posterior hypothalamus, signal was observed in various brain regions, including the tuberomammillary nuclei (*TMN*), cerebral cortex (*CC*), retrosplenial cortex (*RSC*), and field CA3 of the hippocampus (*CA3*). The signal attributed to the *TMN* was verified by *in situ* hybridization for histidine decarboxylase mRNA on separate sections (data not shown). At the level of the anterior pons, the dorsal raphe nuclei (*DRN*), pontine nuclei (*Pn*) and parabigeminal nuclei (*PBG*) displayed specific labeling. This pattern corresponds to that already reported by Malherbe et al. [37] and was in good agreement with the distribution of *Hcrtr2*-expressing neurons previously described [42], [43]. The rats injected with vehicle displayed constant HCRTR2 binding at all time points. In contrast, the animals that received almorexant exhibited a very strong reduction of HCRTR2 radiotracer binding and, 2 h after almorexant injection, no signal could be detected (Figure 7B). Reduction of TB signal was still evident for all brain regions 12 h after almorexant administration.

SB was quantified in the *LC* for HCRTR1 and in 6 brain areas (*TMN*, *CC*, *CA3*, *RSC*, *DRN* and *Pn*) for HCRTR2, and the RO by almorexant was determined for 12 h post-injection (Figure 7C and Figure S7). HCRTR1 RO reached 50–60% from 30 min to 4 h post-injection (maximum: 59% after 2**h) and then returned to basal levels after 6 h. This RO profile paralleled that of almorexant concentration in the plasma (Figure 7D) and brain (Figure S6). For both compartments, drug concentration rose rapidly and reached a peak around 30 min, with plasma levels of 1966.4±349.2 ng/mL and brain levels of 565.8±112.4 ng/g (mean brain/plasma concentration ratio: 0.28). The half-maximal concentrations were achieved between 4 and 6 h.

For HCRTR2, all 6 structures displayed a comparable RO profile (Figure 7C for *DRN* and *TMN*, and Figure S7 for *CC, RSC, Pn* and *CA3*): it was close to 100% within 30 min after dosing, remained at maximal levels at 2 h and 4 h, and started to slowly decline between 4 and 6 h. After 12 h, although the brain and plasma levels of almorexant were strongly reduced (Figure 7C and Figure S6), HCRTR2 occupancy was still elevated with levels between 49 and 67%, depending on brain structure (Figure 7C and Figure S7; *TMN*: 49.2±13.2%; *CC*: 66.1±11.6%; *CA3*∶58.4±11.5%; *RSC*: 64.6±10.7%; *DRN*: 57.7±10.5%; *Pn*: 67.2±13.9%).

## Discussion

This study was undertaken to determine whether blockade of either or both HCRT receptors is more effective in promoting sleep. Multiple dual HCRTR1/R2 antagonists employing different molecular scaffolds have been found to have significant significant sleep-promoting properties [25], [26], [27], [28], [29], [30], [31] Anatomical localization of HCRTRs suggests that both receptors are involved in the promotion of wakefulness [39], [43]. High levels of HCRTR1 are found in *LC* while only HCRTR2 is abundant in the *TMN*. Both receptors are expressed at moderately high levels in the dorsal and medial raphe and in the cholinergic regions of the basal forebrain. In the laterodorsal tegmentum and the pedunculopontine nucleus (brain stem cholinergic regions), the HCRTR1 is predominant. However, some recent reports support the hypothesis that only blockade of the HCRTR2 underlies the hypnotic actions of HCRTR antagonism [30], [31]. Further, one study suggests that antagonism of HCRTR1 attenuates the hypnotic actions of HCRTR2 blockade [32]. Therefore, to help clarify the hypnotic effects of HCRTR blockade, we characterized the pharmacological and pharmacokinetic properties of selective and dual HCRTR antagonists in rat before evaluating their relative efficacy on sleep and wakefulness.

### Pharmacokinetic Considerations

The affinities of almorexant, SB-408124 and SB-334867 at the rat HCRTR1 and HCRTR2 receptors are very similar to those reported for human HCRT receptors (for almorexant, K_i_ values of 4.7 nM and 0.9 nM at hHCRTR1 and hHCRTR2, 37°C, respectively [42]; for SB-334867, K_i_ value of 38.7 nM at rHCRTR1 [34]; for SB-408124, K_i_ value of 26.9 nM at rHCRTR1 [34]). Almorexant had high affinity for both HCRTRs and displayed a slow rate of dissociation from rHCRTR2 membranes *in vitro*, which translated into a long-lasting occupancy of the HCRTR2 *in vivo*. This property likely underlies some of the pharmacological effects described here. Among the three antagonists tested, almorexant had the highest systemic plasma clearance, highest Vss but lowest oral bioavailability; both SB-334867 and SB-408124 had low clearances and medium to low bioavailability. Importantly, SB-408124 had a very low free fraction and was found to penetrate the brain poorly, especially when compared to the other compounds. This prompted us to use SB-334867 for evaluating the effects of selective HCRTR1 blockade on sleep.

### Effects of Selective HCRTR1 and HCRTR2 Antagonists on Sleep/wake

Selective blockade of HCRTR2 clearly results in sleep promotion. The HCRTR2 antagonist JNJ-10397049 reduced NR latency during both the light and dark phases, increased NR duration in the light phase, and increased both NR and REM duration during the dark phase [30], [31]. Here, although EMPA had no effect on either NR or REM latency when administered in the mid-dark phase, it increased cumulative NR for the first 4 and 6 h. Conversely, icv infusion of an HCRTR2 agonist, [Ala^11^]orexin-B, during the light period dose-dependently increased wake duration and decreased the amounts of both NR and REM sleep [44]. The effects of HCRT1 (orexin-A) on wakefulness and NREM sleep were reduced more in *OX2R* ^−/−^ mice than in *OX1R* ^−/−^ mice, implying that HCRTR2 has a greater influence than HCRTR1 on these parameters, at least in mice [45].

The selective HCRTR1 antagonist SB-334867 dose-dependently reduced LMA and, at 3 and 30 mg/kg i.p., increased cumulative NR for the first 4 and 6 h. These results differ from those of Dugovic *et al.* [32] who reported that selective blockade of HCRTR1 using SB-408124 had no effect on sleep, although it reduced LMA. However, the time of drug administration differed between these studies (middle vs. start of the active phase). By the middle of the active phase, both endogenous HCRT tone [46], [47] and sleep pressure are increased, so HCRTR antagonists are more likely to be effective at this time of day than at dark onset.

A previous study showed that SB-334867 blocked HCRT1-induced effects on REM sleep but did not alter any sleep parameters when administered alone [36]. However, only the first hour after treatment was examined whereas, here, effects of SB-334867 on sleep were only apparent after 2 h. Importantly, we showed that SB-408124 exhibits poor pharmacokinetic properties, with notably low free fraction and little brain penetration, which likely limits its *in vivo* efficacy. The brain-to-plasma ratio for SB-408124 is 0.03, which is in the range of blood contamination levels obtained with the residual blood carried over in the brain homogenate (in the absence of compound in the brain). Although Dugovic *et al.* [32] did not specifically report brain-to-plasma ratios, they did report both brain and plasma concentrations following administration of SB-408124 at 30 mg/kg. Using these numbers, a brain-to-plasma ratio for SB-408124 is calculated to be 0.012 (using C_max_ values given in text: brain-to-plasma ratio = 1.09/84.29 = 0.012), which is in good agreement with our findings. This observation most likely explains why Dugovic *et al.* [32] did not detect effect on sleep. There are numerous examples of compounds lacking central efficacy due to insufficient brain exposure. For example, the reduced ability of second-generation H1 anti-histaminic drugs to cross the blood-brain barrier (BBB) as compared to the first generation of drugs, prevents them from causing centrally-mediated side effects such as sedation [48], [49], [50]. Similarly, the antidiarrheal medication loperamide is a potent agonist of the µ opiate receptor that is devoid of opioid central effects at usual doses in patients [51]. This directly results from the low brain exposure caused by the P-glycoprotein (P-gp) transporter at the BBB [51]. Administration of the drug to P-gp-deficient mice or co-administration with a P-gp blocker both increase brain levels and trigger central effects typically observed with brain penetrant opioids, such as analgesia [52], [53] or respiratory depression [54]. Our observation made with SB-408124 underscores that verification of brain penetration is a prerequisite for the conception and use of centrally-acting drugs [55], [56].

On the other hand, it is difficult to reconcile the poor brain penetration of SB-408124, both documented here and also evident in the study of Dugovic *et al.* (estimation: 0.012), with some indications of central localization following subcutaneous administration of 30 mg/kg, i.e. the 90% HCRTR1 occupancy observed in the *tenia tecta* and the SB-408124-mediated elevation of extracellular dopamine levels in the prefrontal cortex [32]. A heterogeneous distribution of the drug is unlikely, and further experiments will be necessary to delineate more precisely the free concentration of the compound, such as microdialysis studies and measures of binding to brain tissue homogenates.

### Dual HCRTR Antagonists as Potential Hypnotic Medications

Dual HCRTR1/R2 antagonists are now well-established to induce sleep. In rats, almorexant administered po at the beginning of the dark phase promoted both NR and REM sleep and, at a higher dose, reduced NR and REM latency [27]. The effects on sleep duration but not sleep latency were confirmed when almorexant was administered sc [32]. Here, we report that almorexant given ip at the mid-dark phase also increases sleep duration. However, in contrast to Dugovic *et al*., we found that almorexant at 30 and 100 mg/kg reduced NR latency and the 30 mg/kg dose also decreased REM latency. These differences likely reflect the greater sensitivity of the sleep/wake bioassay when injections occur in the mid-dark period after a sleep debt has accumulated. Recently, other dual HCRTR1/R2 antagonists have also been reported to reduce active wake and increase both NR or delta sleep and REM sleep when administered near the mid-dark phase [25], [26], [27], [28], [29], [30], [31], [57]. Thus, multiple HCRTR1/R2 antagonists seem to be effective in inducing sleep.

Our results indicate some promising aspects of HCRT antagonists as hypnotic agents. First, in contrast to current hypnotics such as zolpidem which increase NR and suppress REM sleep, none of the three HCRTR antagonists affected the REM:NR ratio, indicating that both REM and NR increased proportionally. Second, in comparison to zolpidem, HCRTR antagonists only triggered a limited, physiological reduction of body temperature. Lastly, no excess wakefulness was observed during the subsequent light period. A proportional increase of REM and NR sleep without rebound wakefulness and a mild change in core temperature are desirable properties of substances that induce “physiological” sleep.

On the other hand, the mechanism by which these HCRTR antagonists increased sleep duration suggests disruption of normal sleep/wake architecture. SB-334867 increased NR through a combination of small increases in both the number and duration of NR bouts that, although not significant for any particular hour, cumulatively summated into an overall significant NR increase at 4 and 6 h. For EMPA, a greater number of NR bouts underlie the overall increase in NR at the highest dose. For almorexant, NR augmentation resulted from an increased number of NR bouts without a change in bout duration, confirming previous results [32]. The increase in NR, however, was also associated with greater numbers of both W and REM bouts, particularly at the highest dose examined. Thus, although almorexant produces an overall increase in NR sleep that is greater than the other HCRTR antagonists, this is achieved through a fragmented sleep architecture. In this regard, almorexant-treated rats appear somewhat similar to *orexin* null mutant [4] or *orexin/ataxin-3* [12] mice which have disrupted sleep architecture (although these strains also exhibit cataplexy). However, the fragmentation of sleep architecture induced by dual HCRTR antagonists is consistent with the concept that the HCRT system stabilizes arousal states and minimizes the number of transitions between states [58]. Since drugs were administered to healthy animals during their active period, a more fragmented sleep architecture would be predicted. Rather than driving sleep *per se*, HCRTR antagonism seems to create a permissive neural environment for sleep to occur. Since the drive for sleep was low at the time of administration, more frequent sleep bouts without increases in bout durations could be expected.

**Figure 8:**
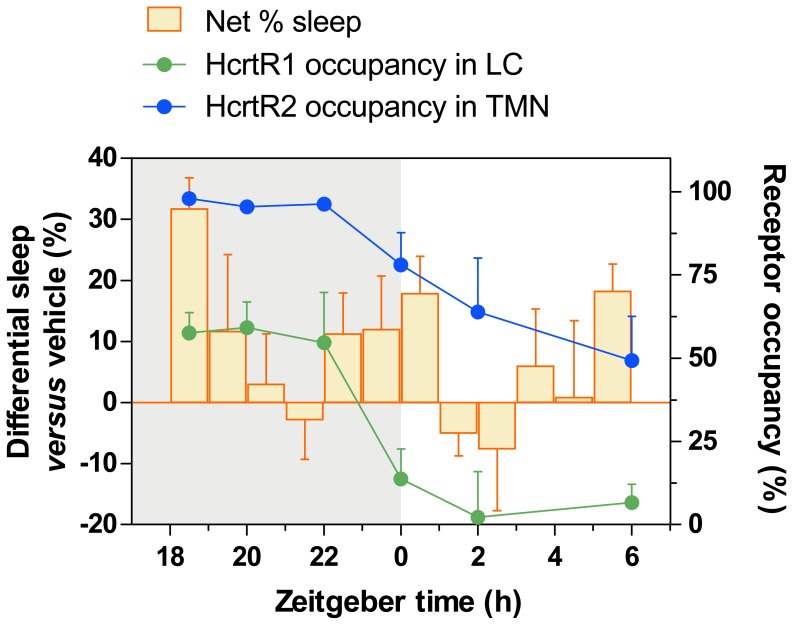
Net effect of almorexant on the percentage of sleep compared to HCRTR1 and HCRTR2 occupancies. The percentage of total sleep (%NR + %REM) in the vehicle-injected animals was subtracted from that of almorexant-treated rats (30 mg/kg) and was plotted over time. HCRTR1 occupancy in the locus coeruleus (*LC)* and HCRTR2 occupancy in the tuberomammillary nuclei (*TMN*) are shown in parallel. Injection occurred at ZT18. *Gray area,* dark phase; *White area*, light phase.

### Absence of Cataplexy but Facilitation of REM Sleep

One concern regarding the development of HCRTR antagonists is the possibility of inducing cataplexy as occurs in *HcrtR2* mutant dogs [3] or *HcrtR2* null mutant mice [59]. In the present study, we saw no evidence of cataplexy produced by any of the three compounds, even at the highest dose tested. However, almorexant significantly increased REM bout duration during the first hour after treatment and the highest dose – which presumably resulted in the most complete HCRTR blockade – produced 2 to 3 fold as many REM bouts during the latter half of the dark period when compared to vehicle. These observations indicate that HCRTR antagonism facilitates REM sleep occurrence, as noted by others [59].

### Relationship between HCRTR Occupancy and Sleep

Whereas 30 mg/kg ip almorexant resulted in approximately 50% HCRTR1 occupancy, HCRTR2 occupancy was nearly complete in brain regions important for sleep/wake control. Moreover, while HCRTR1 occupancy declined after 4 h, HCRTR2 occupancy remained high even 12 h after treatment. While our results for HCRTR2 are consistent with a previous report, those for HCRTR1 differ [32]. A primary difference between these studies is the brain location used for determination of HCRTR1 occupancy: whereas Dugovic *et al.* used the *tenia tecta*, we measured HCRTR1 occupancy in the *LC*, an area implicated in sleep/wake control.

Figure 8 correlates RO with the net amount of sleep induced by almorexant at 30 mg/kg compared to vehicle. Since HCRTR2 occupancy is virtually 100% following this dose of almorexant while HCRTR1 occupancy is ∼50%, it is likely that the stronger sleep-promoting effects observed at 100 mg/kg are due to greater HCRTR1 blockade. Figure 8 demonstrates that the sleep-promoting effects of almorexant do not simply mirror the RO data. The greatest amount of sleep occurred in the first hour after almorexant administration when occupancy of HCRTRs was maximal. Surprisingly, despite elevated occupancy of HCRTRs in subsequent hours, the hypnotic effect dissipated, suggesting that other arousal-promoting systems can overcome HCRTR blockade and produce wakefulness. In contrast, near the end of the dark phase when sleep pressure is elevated, partial HCRTR blockade was sufficient to produce sleep. These data highlight the contrasting sleep-promoting mechanisms between HCRTR antagonists and other hypnotic medications such as zolpidem. Whereas the latter compounds trigger long-lasting sleep and affect sleep intensity (sleep-inducing effect), HCRTR antagonists seem to merely antagonize wakefulness, generating conditions that allow sleep to occur (sleep permissive action).

### Conclusion

Our results support the hypothesis that dual HCRTR1/R2 blockade is more effective in promoting sleep than selective blockade of either HCRTR alone. A similar conclusion was reached in a recent study of HCRT receptor knockout mice [45]. Although both HCRTR1 (SB-334867) and HCRTR2 (EMPA) antagonists produced somnogenic effects, neither promoted sleep to the levels of the dual HCRTR antagonist almorexant. Furthermore, since the lowest doses of almorexant that were sleep-promoting (30 mg/kg) bind virtually 100% of the HCRTR2s while only 50% of the HCRTR1s are occupied at that dose, the stronger sleep-promoting effects of higher doses are likely due to additional blockade of HCRTR1. These data support the notion that HCRTR antagonists are a promising avenue for sleep/wake therapeutics, with the qualifications stated above. However, given the involvement of the HCRT system in many physiological functions [9], [60] including respiratory control [61], [62], [63], [64], careful screening for side effects of HCRTR antagonists will be needed.

## Supporting Information

Figure S1 **Chemical structures of the compounds used in this study.** Receptor selectivity is indicated into parentheses. All compounds except zolpidem are selective HCRTR antagonists. Zolpidem is a gama-aminobutyric acid (GABA) A-receptor agonist.
(TIF)

Figure S2 **Hourly delta power normalized to the 24 h average vehicle control. A:** 3 concentrations of SB-334867 vs. ZOL and vehicle. ANOVA is significant for treatment by time only (F = 3.80, p<0.0001). For treatment by time: **ZT19:** SB-334867 at 3 mg/kg > vehicle; ZOL >334867 at 3 and 10 mg/kg and vehicle. **ZT24:** 334867 at 3 and 10 mg/kg > ZOL; Vehicle >334867 at 10 and 30 mg/kg and ZOL **B:** 3 concentrations of EMPA vs. ZOL and vehicle. ANOVA is significant for treatment (see legend, F = 13.47, p<0.0001) and for treatment by time (F = 11.86, p<0.0001). For treatment by time: **ZT19:** ZOL > all other conditions. **ZT20:** ZOL > all other conditions. **ZT21:** EMPA at 30 mg/kg > vehicle; ZOL > EMPA at 100 mg/kg and vehicle. **ZT22:** EMPA at 30 mg/kg > vehicle. **ZT23:** EMPA at 10 mg/kg > ZOL. **C:** 3 concentrations of almorexant vs. ZOL and vehicle. ANOVA is significant for treatment by time only (F = 2.63, p = 0.0005). For treatment by time: **ZT20:** Vehicle > almorexant at 100 mg/kg. **ZT23:** Almorexant at 10 mg/kg > vehicle. **ZT24:** Vehicle > almorexant at 100 mg/kg.
(TIF)

Figure S3 **Hourly distribution of Wake Bout Duration and the Number of Wake Bouts.** Wake Bout Duration (left) and Number of Wake Bouts (right) for 6 h prior to and 18 h after administration of SB-334867 (**A**), EMPA (**B**), and almorexant (**C**) as compared to zolpidem (ZOL). Shaded area represents the dark phase; vertical dotted line shows the first h following injection. **A:** The Wake Bout Duration for 3 concentrations of SB 334867 vs. ZOL and vehicle. No significant differences were found. **A’:** The Wake Bout Number for 3 concentrations of SB 334867 vs. ZOL and vehicle. ANOVA for ZT1-ZT6 is significant for treatment by time (F = 1.82, p = 0.02341). For treatment by time: **ZT2:** SB 334867 at 10 mg/kg and vehicle < ZOL vehicle < SB 334867 at 30 mg/kg **ZT4:** SB 334867 at 30 mg/kg and ZOL < vehicle **B:** The Wake Bout Duration for 3 concentrations of EMPA vs. ZOL and vehicle. No ANOVA’s were significant. **B’:** The Wake Bout Number for 3 concentrations of EMPA vs. ZOL and vehicle. ANOVA for ZT19-ZT24 is significant for treatment (F = 3.65, p = 0.01350). ANOVA for ZT7-ZT12 is significant for treatment (F = 4.24, p = 0.00647) For treatment by time: **ZT19:** vehicle < ZOL **ZT20:** vehicle < EMPA at 30 mg/kg **ZT22:** vehicle < ZOL **ZT24:** vehicle < EMPA at 10, 30 and 100 mg/kg **ZT7:** EMPA at 10 mg/kg < ZOL **ZT11:** vehicle < ZOL **C:** The Wake Bout Duration for 3 concentrations of Almorexant vs. ZOL and vehicle. ANOVA for ZT19-ZT24 is significant for treatment (F = 4.01, p = 0.01077) and for treatment by time (F = 2.32, p = 0.00234). For treatment by time: **ZT20:** Almorexant at 100 mg/kg < ZOL **ZT21:** Almorexant at 30 and 100 mg/kg < ZOL **ZT22:** Almorexant at 100 mg/kg < ZOL and vehicle **C’:** The Wake Bout Number for 3 concentrations of Almorexant vs. ZOL and vehicle. ANOVA for ZT19-ZT24 is significant for treatment (F = 8.82, p = 0.00001) and for treatment by time (F = 2.07, p = 0.00769). ANOVA for ZT7-ZT12 is significant for treatment (F = 3.39, p = 0.02208). For treatment by time: **ZT19:** vehicle < Almorexant at 30 and 100 mg/kg **ZT20:** ZOL < Almorexant at 10, 30 and 100 mg/kg **ZT21:** ZOL < Almorexant at 30 and 100 mg/kg **ZT22:**ZOL and vehicle < Almorexant at 100 mg/kg **ZT23:** vehicle < Almorexant at 100 mg/kg **ZT24:** vehicle < Almorexant at 100 mg/kg **ZT9:** Almorexant at 10 and 30 mg.kg < vehicle.
(TIF)

Figure S4 **Hourly distribution of NR Bout Duration and Number of NR Bouts.** NR Bout Duration (left) and Number of NR Bouts (right) for 6 h prior to and 18 h after administration of SB-334867 (**A**), EMPA (**B**), and almorexant (**C**) as compared to zolpidem (ZOL). Shaded area represents the dark phase; vertical dotted line shows the first h following injection. **A:** The NR Bout Duration for 3 concentrations of SB 334867 vs. ZOL and vehicle. ANOVA for ZT19-ZT24 is significant for treatment (F = 12.46, p<0.00001) and for treatment by time (F = 4.57, p<0.00001). ANOVA for ZT1-ZT6 is significant for treatment (F = 4.70, p = 0.00498) and for treatment by time (F = 3.16, p = 0.00004). For treatment by time: **ZT19:** SB 334867 at 3 mg/kg and vehicle < ZOL **ZT20:** all other conditions < ZOL **ZT21:** vehicle < SB 334867 at 30 mg/kg and ZOL **ZT24:** vehicle < SB 334867 at 3 mg/kg **ZT1:** ZOL < SB 334867 at 3 and 10 mg/kg and vehicle SB 334867 at 3 mg/kg < vehicle **ZT3:** SB 334867 at 30 mg/kg and ZOL < vehicle **A’:** The NR Bout Number for 3 concentrations of SB 334867 vs. ZOL and vehicle. ANOVA for ZT1-ZT6 is significant for treatment by time (F = 1.81, p = 0.02532). For treatment by time: **ZT1:** vehicle < SB 334867 at 3 and 30 mg/kg and ZOL **ZT4:** SB 334867 at 3 mg/kg < vehicle **B:** The NR Bout Duration for 3 concentrations of EMPA vs. ZOL and vehicle. ANOVA for ZT19-ZT24 is significant for treatment (F = 13.46, p<0.00001) and for treatment by time (F = 5.34, p<0.00001). ANOVA for ZT1-ZT6 is significant for treatment (F = 7.99, p = 0.00010). ANOVA for ZT7-ZT12 is significant for treatment (F = 3.03, p = 0.02981). For treatment by time: **ZT19:** all other conditions < ZOL **ZT20:** all other conditions < ZOL **ZT23:** ZOL < EMPA at 10 mg/kg **ZT24:** ZOL < EMPA at 30 mg/kg **ZT2:** ZOL < EMPA at 30 mg/kg **ZT3:** ZOL < EMPA at 10 and 100 mg/kg and vehicle **ZT5:** ZOL < EMPA at 10 and 100 mg/kg and vehicle EMPA at 30 and 100 mg/kg < vehicle **ZT6:** ZOL < EMPA at 10 mg/kg and vehicle EMPA at 100 mg/kg < vehicle **B’:** The NR Bout Number for 3 concentrations of EMPA vs. ZOL and vehicle. No ANOVA’s were significant. **C:** The NR Bout Duration for 3 concentrations of Almorexant vs. ZOL and vehicle. ANOVA for ZT19-ZT24 is significant for treatment (F = 16.44, p<0.00001) and for treatment by time (F = 5.34, p<0.00001). ANOVA for ZT1-ZT6 is significant for treatment (F = 4.83, p = 0.00433) and for treatment by time (F = 2.24, p = 0.00341). For treatment by time: **ZT19:** all other conditions < ZOL vehicle < Almorexant at 100 mg/kg **ZT20:** all other conditions < ZOL **ZT21:** all other conditions < ZOL **ZT22:** Almorexant at 10 and 30 mg/kg < ZOL **ZT2:** ZOL < Almorexant at 10 and 30 mg/kg and vehicle Almorexant at 100 mg/kg < vehicle **ZT3:** ZOL < Almorexant at 10 mg/kg **ZT4:** ZOL < Almorexant at 10 mg/kg **ZT5:** ZOL < Almorexant at 10 mg/kg **ZT6:** ZOL < Almorexant at 30 mg/kg **C’:** The NR Bout Number for 3 concentrations of Almorexant vs. ZOL and vehicle. ANOVA for ZT19-ZT24 is significant for treatment (F = 12.58, p<0.00001) and for treatment by time (F = 2.41, p = 0.00149). ANOVA for ZT1-ZT6 is significant for treatment (F = 4.18, p = 0.00890). For treatment by time: **ZT19:** vehicle < Almorexant at 30 and 100 mg/kg **ZT20:** ZOL < Almorexant at 10, 30 and 100 mg/kg vehicle < Almorexant at 100 mg/kg **ZT21:** ZOL < Almorexant at 10, 30 and 100 mg/kg **ZT22:** ZOL and vehicle < Almorexant at 100 mg/kg **ZT23:** vehicle < Almorexant at 100 mg.kg **ZT24:** vehicle < Almorexant at 100 mg.kg **ZT1:** vehicle < ZOL.
(TIF)

Figure S5 **Hourly distribution of REM Sleep Bout Duration and the Number of REM Sleep Bouts.** REM Sleep Bout Duration (left) and the Number of REM Sleep Bouts (right) for 6 h prior to and 18 h after administration of SB-334867 (**A**), EMPA (**B**), and almorexant (**C**) as compared to zolpidem (ZOL). Shaded area represents the dark phase; vertical dotted line shows the first h following injection. **A:** The REM Bout Duration for 3 concentrations of SB 334867 vs. ZOL and vehicle. ANOVA for ZT19-ZT24 is significant for treatment (F = 4.40, p = 0.00692) and treatment by time (F = 2.16, p = 0.00500). For treatment by time: **ZT19:** ZOL < SB 334867 at 3 mg/kg **ZT20:** ZOL < all other conditions **ZT23:** vehicle < all other conditions **ZT24:** SB 334867 at 10**mg/kg < ZOL vehicle < SB 334867 at 3 mg/kg **A’:** The REM Bout Number for 3 concentrations of SB 334867 vs. ZOL and vehicle. ANOVA for ZT19-ZT24 is significant for treatment by time only (F = 4.49, p = 0.00625). For treatment by time: **ZT20:** ZOL < all other conditions **ZT24:** vehicle < SB 334867 at 30 mg/kg **B:** The REM Bout Duration for 3 concentrations of EMPA vs. ZOL and vehicle. ANOVA for ZT19-ZT24 is significant for treatment by time (F = 1.71, p = 0.03515). ANOVA for ZT1-ZT6 is significant for treatment (F = 4.88, p = 0.00015) and for treatment by time (F = 2.81, p = 0.00015). For treatment by time: **ZT21:** ZOL < EMPA at 100 mg/kg **ZT24:** EMPA at 100 mg/kg < vehicle **ZT1:** EMPA at 100 mg/kg < ZOL all other conditions < vehicle **ZT4:** EMPA at 10 and 30 mg/kg < vehicle **ZT5:** ZOL < EMPA at 10 mg/kg **B’:** The REM Bout Number for 3 concentrations of EMPA vs. ZOL and vehicle. ANOVA for ZT19-ZT24 is significant for treatment (F = 3.99, p = 0.00888) and for treatment by time (F = 1.96, p = 0.01112). For treatment by time: **ZT20:** ZOL < all other conditions **ZT22:** vehicle < ZOL **ZT23:** ZOL < vehicle **C:** The REM Bout Duration for 3 concentrations of Almorexant vs. ZOL and vehicle. ANOVA for ZT19-ZT24 is significant for treatment by time (F = 6.91, p<0.00001). ANOVA for ZT1-ZT6 is significant for treatment (F = 4.45, p = 0.00657). For treatment by time: **ZT19:** ZOL and vehicle < Almorexant at 10, 30 and 100 mg/kg **ZT20:** all other conditions < ZOL **ZT24:**ZOL < Almorexant at 10 and 100 mg/kg and vehicle Almorexant at 30 mg/kg < vehicle **C’:** The REM Bout Number for 3 concentrations of Almorexant vs. ZOL and vehicle. ANOVA for ZT19-ZT24 is significant for treatment (F = 9.29, p = 0.00007) and for treatment by time (F = 2.96, p = 0.00010). For treatment by time: **ZT19:** ZOL and vehicle < Almorexant at 30 mg/kg **ZT20:** ZOL < Almorexant at 10 and 30 mg/kg and vehicle **ZT21:** ZOL < all other conditions **ZT22:** ZOL and vehicle < Almorexant at 100 mg/kg **ZT23:** vehicle < Almorexant at 100 mg/kg **ZT24:** ZOL and vehicle < Almorexant at 100 mg/kg.
(TIF)

Figure S6 **Brain concentration of almorexant.** Time course of almorexant concentration in the brain of rats injected intraperitoneally with 30 mg/kg at the mid-dark phase (same animals as in Figures 7). Data are the mean±SEM (n = 5 rats per group).
(PDF)

Figure S7 **HCRTR2 occupancy in the cerebral cortex, retrosplenial cortex, pontine nuclei, and hippocampus.** Data are the mean±SEM (n = 5 rats per group). *, *p*<0.001 vs. time 0; ##, *p*<0.01, #, *p*<0.05 vs. time 30 min (one-way ANOVA followed by Dunnett’s analysis). Almorexant plasma concentrations (data from Figure 7) are shown for comparison.
(TIF)

Materials and Methods S1 **Expanded materials and methods for both** ***in vitro*** **and** ***in vivo*** **experiments as referenced in the text.**
(DOCX)

Text S1 **Expanded legends for** **Figures 5** **and** **6** **that include detailed statistical results.**
(DOCX)

Table S1 **Pharmacokinetic assessment of almorexant, SB-334867 and SB-408124 after i.v. and p.o. administration to Wistar rat.**
(DOCX)

Table S2 **CEREP selectivity screen in the broad radioligand binding assays were undertaken to determine the pharmacological activity of SB-334867.**
(DOCX)

Table S3 **Measures of state consolidation for 6 h following the administration of SB-334867.**
(DOCX)

Table S4 **Measures of state consolidation for 6 h following the administration of EMPA.**
(DOCX)

Table S5 **Measures of state consolidation for 6 h following the administration of almorexant.**
(DOCX)
