# Supplementary figures and images for: Dual Hypocretin Receptor Antagonism Is More Effective for Sleep Promotion than Antagonism of Either Receptor Alone
Source: PLoS One. 2012 Jul 2;7(7):e39131. doi: 10.1371/journal.pone.0039131 (PMC3388080; doi:10.1371/journal.pone.0039131)

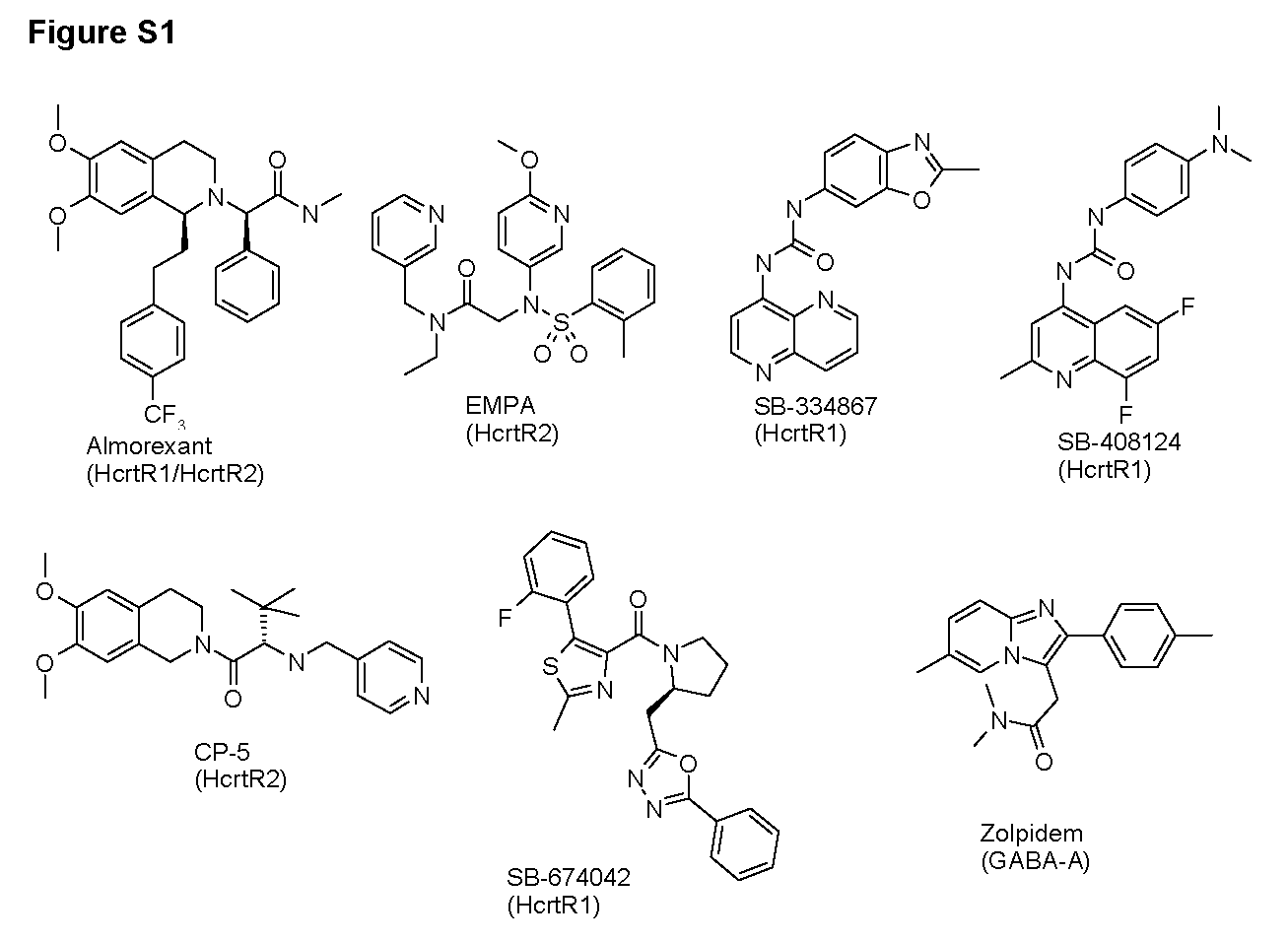

Supplement: Figure S1 — Chemical structures of the compounds used in this study. Receptor selectivity is indicated into parentheses. All compounds except zolpidem are selective HCRTR antagonists. Zolpidem is a gama-aminobutyric acid (GABA) A-receptor agonist. (TIF) [file pone.0039131.s001.tif]

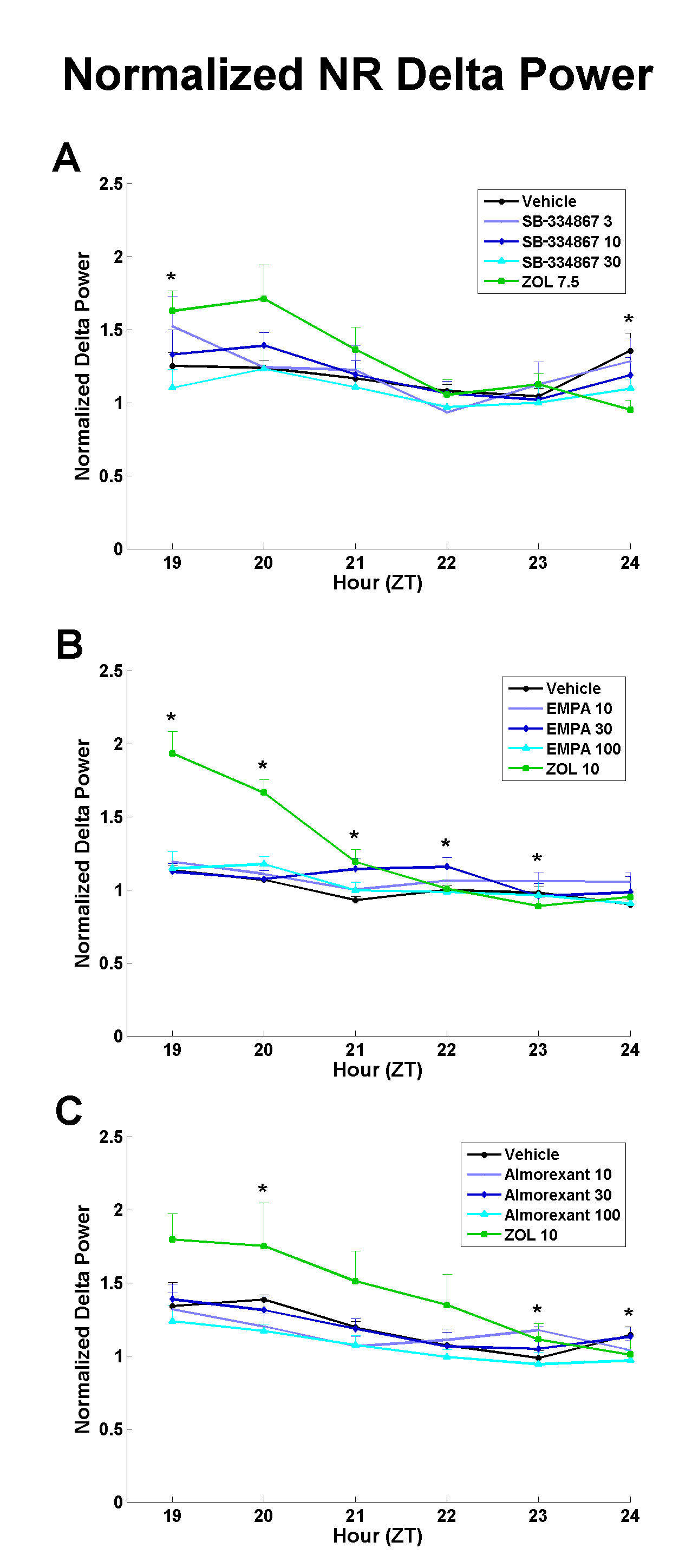

Supplement: Figure S2 — Hourly delta power normalized to the 24 h average vehicle control. A: 3 concentrations of SB-334867 vs. ZOL and vehicle. ANOVA is significant for treatment by time only (F = 3.80, p<0.0001). For treatment by time: ZT19: SB-334867 at 3 mg/kg > vehicle; ZOL >334867 at 3 and 10 mg/kg and vehicle. ZT24: 334867 at 3 and 10 mg/kg > ZOL; Vehicle >334867 at 10 and 30 mg/kg and ZOL B: 3 concentrations of EMPA vs. ZOL and vehicle. ANOVA is significant for treatment (see legend, F = 13.47, p<0.0001) and for treatment by time (F = 11.86, p<0.0001). For treatment by time: ZT19: ZOL > all other conditions. ZT20: ZOL > all other conditions. ZT21: EMPA at 30 mg/kg > vehicle; ZOL > EMPA at 100 mg/kg and vehicle. ZT22: EMPA at 30 mg/kg > vehicle. ZT23: EMPA at 10 mg/kg > ZOL. C: 3 concentrations of almorexant vs. ZOL and vehicle. ANOVA is significant for treatment by time only (F = 2.63, p = 0.0005). For treatment by time: ZT20: Vehicle > almorexant at 100 mg/kg. ZT23: Almorexant at 10 mg/kg > vehicle. ZT24: Vehicle > almorexant at 100 mg/kg. (TIF) [file pone.0039131.s002.tif]

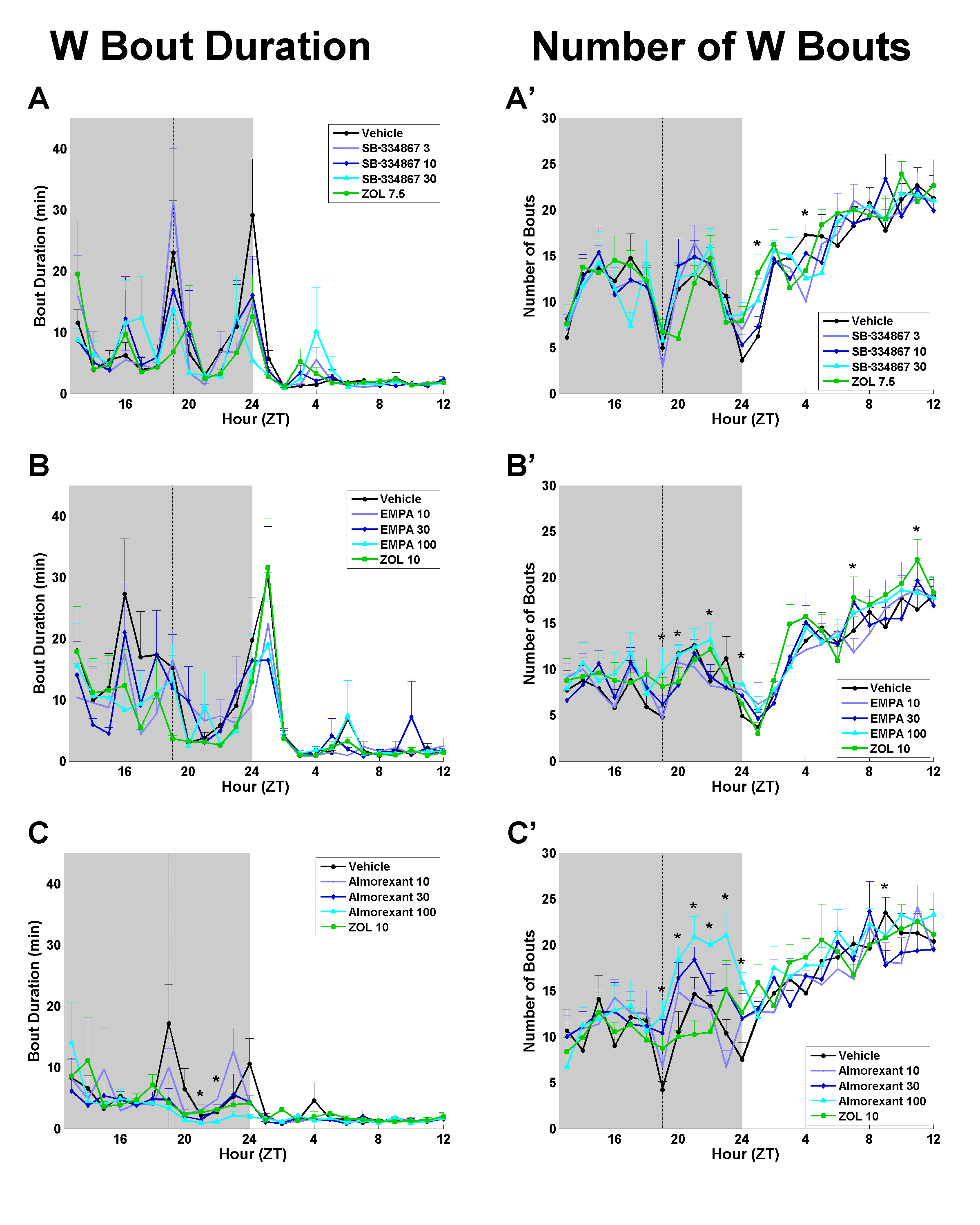

Supplement: Figure S3 — Hourly distribution of Wake Bout Duration and the Number of Wake Bouts. Wake Bout Duration (left) and Number of Wake Bouts (right) for 6 h prior to and 18 h after administration of SB-334867 (A), EMPA (B), and almorexant (C) as compared to zolpidem (ZOL). Shaded area represents the dark phase; vertical dotted line shows the first h following injection. A: The Wake Bout Duration for 3 concentrations of SB 334867 vs. ZOL and vehicle. No significant differences were found. A’: The Wake Bout Number for 3 concentrations of SB 334867 vs. ZOL and vehicle. ANOVA for ZT1-ZT6 is significant for treatment by time (F = 1.82, p = 0.02341). For treatment by time: ZT2: SB 334867 at 10 mg/kg and vehicle < ZOL vehicle < SB 334867 at 30 mg/kg ZT4: SB 334867 at 30 mg/kg and ZOL < vehicle B: The Wake Bout Duration for 3 concentrations of EMPA vs. ZOL and vehicle. No ANOVA’s were significant. B’: The Wake Bout Number for 3 concentrations of EMPA vs. ZOL and vehicle. ANOVA for ZT19-ZT24 is significant for treatment (F = 3.65, p = 0.01350). ANOVA for ZT7-ZT12 is significant for treatment (F = 4.24, p = 0.00647) For treatment by time: ZT19: vehicle < ZOL ZT20: vehicle < EMPA at 30 mg/kg ZT22: vehicle < ZOL ZT24: vehicle < EMPA at 10, 30 and 100 mg/kg ZT7: EMPA at 10 mg/kg < ZOL ZT11: vehicle < ZOL C: The Wake Bout Duration for 3 concentrations of Almorexant vs. ZOL and vehicle. ANOVA for ZT19-ZT24 is significant for treatment (F = 4.01, p = 0.01077) and for treatment by time (F = 2.32, p = 0.00234). For treatment by time: ZT20: Almorexant at 100 mg/kg < ZOL ZT21: Almorexant at 30 and 100 mg/kg < ZOL ZT22: Almorexant at 100 mg/kg < ZOL and vehicle C’: The Wake Bout Number for 3 concentrations of Almorexant vs. ZOL and vehicle. ANOVA for ZT19-ZT24 is significant for treatment (F = 8.82, p = 0.00001) and for treatment by time (F = 2.07, p = 0.00769). ANOVA for ZT7-ZT12 is significant for treatment (F = 3.39, p = 0.02208). For treatment by time: ZT19: vehicle < Almorexant at 30 and 100 mg/kg Z [file pone.0039131.s003.tif]

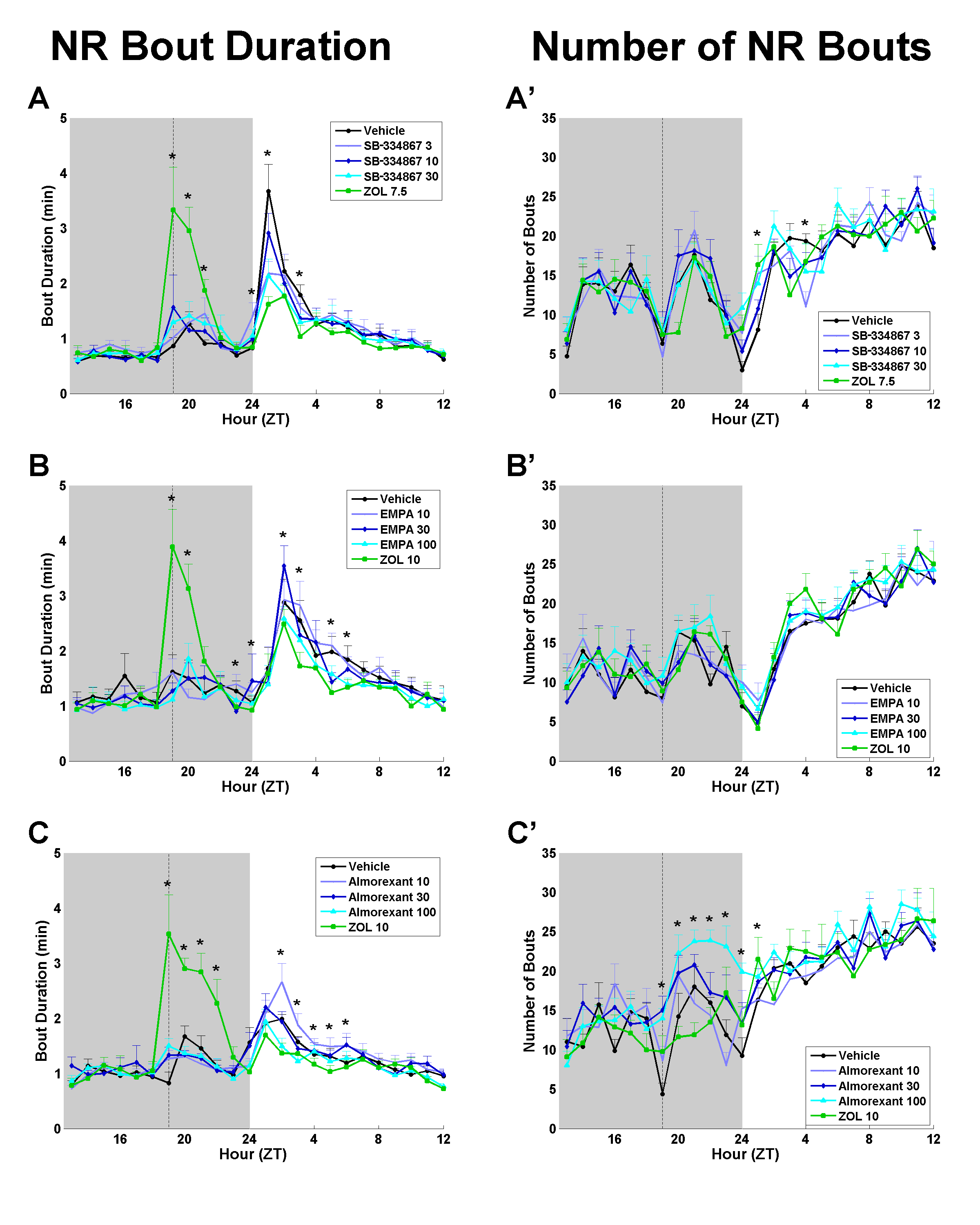

Supplement: Figure S4 — Hourly distribution of NR Bout Duration and Number of NR Bouts. NR Bout Duration (left) and Number of NR Bouts (right) for 6 h prior to and 18 h after administration of SB-334867 (A), EMPA (B), and almorexant (C) as compared to zolpidem (ZOL). Shaded area represents the dark phase; vertical dotted line shows the first h following injection. A: The NR Bout Duration for 3 concentrations of SB 334867 vs. ZOL and vehicle. ANOVA for ZT19-ZT24 is significant for treatment (F = 12.46, p<0.00001) and for treatment by time (F = 4.57, p<0.00001). ANOVA for ZT1-ZT6 is significant for treatment (F = 4.70, p = 0.00498) and for treatment by time (F = 3.16, p = 0.00004). For treatment by time: ZT19: SB 334867 at 3 mg/kg and vehicle < ZOL ZT20: all other conditions < ZOL ZT21: vehicle < SB 334867 at 30 mg/kg and ZOL ZT24: vehicle < SB 334867 at 3 mg/kg ZT1: ZOL < SB 334867 at 3 and 10 mg/kg and vehicle SB 334867 at 3 mg/kg < vehicle ZT3: SB 334867 at 30 mg/kg and ZOL < vehicle A’: The NR Bout Number for 3 concentrations of SB 334867 vs. ZOL and vehicle. ANOVA for ZT1-ZT6 is significant for treatment by time (F = 1.81, p = 0.02532). For treatment by time: ZT1: vehicle < SB 334867 at 3 and 30 mg/kg and ZOL ZT4: SB 334867 at 3 mg/kg < vehicle B: The NR Bout Duration for 3 concentrations of EMPA vs. ZOL and vehicle. ANOVA for ZT19-ZT24 is significant for treatment (F = 13.46, p<0.00001) and for treatment by time (F = 5.34, p<0.00001). ANOVA for ZT1-ZT6 is significant for treatment (F = 7.99, p = 0.00010). ANOVA for ZT7-ZT12 is significant for treatment (F = 3.03, p = 0.02981). For treatment by time: ZT19: all other conditions < ZOL ZT20: all other conditions < ZOL ZT23: ZOL < EMPA at 10 mg/kg ZT24: ZOL < EMPA at 30 mg/kg ZT2: ZOL < EMPA at 30 mg/kg ZT3: ZOL < EMPA at 10 and 100 mg/kg and vehicle ZT5: ZOL < EMPA at 10 and 100 mg/kg and vehicle EMPA at 30 and 100 mg/kg < vehicle ZT6: ZOL < EMPA at 10 mg/kg and vehicle EMPA at 100 mg/kg < vehicle B’: The NR Bout Number for 3 concentration [file pone.0039131.s004.tif]

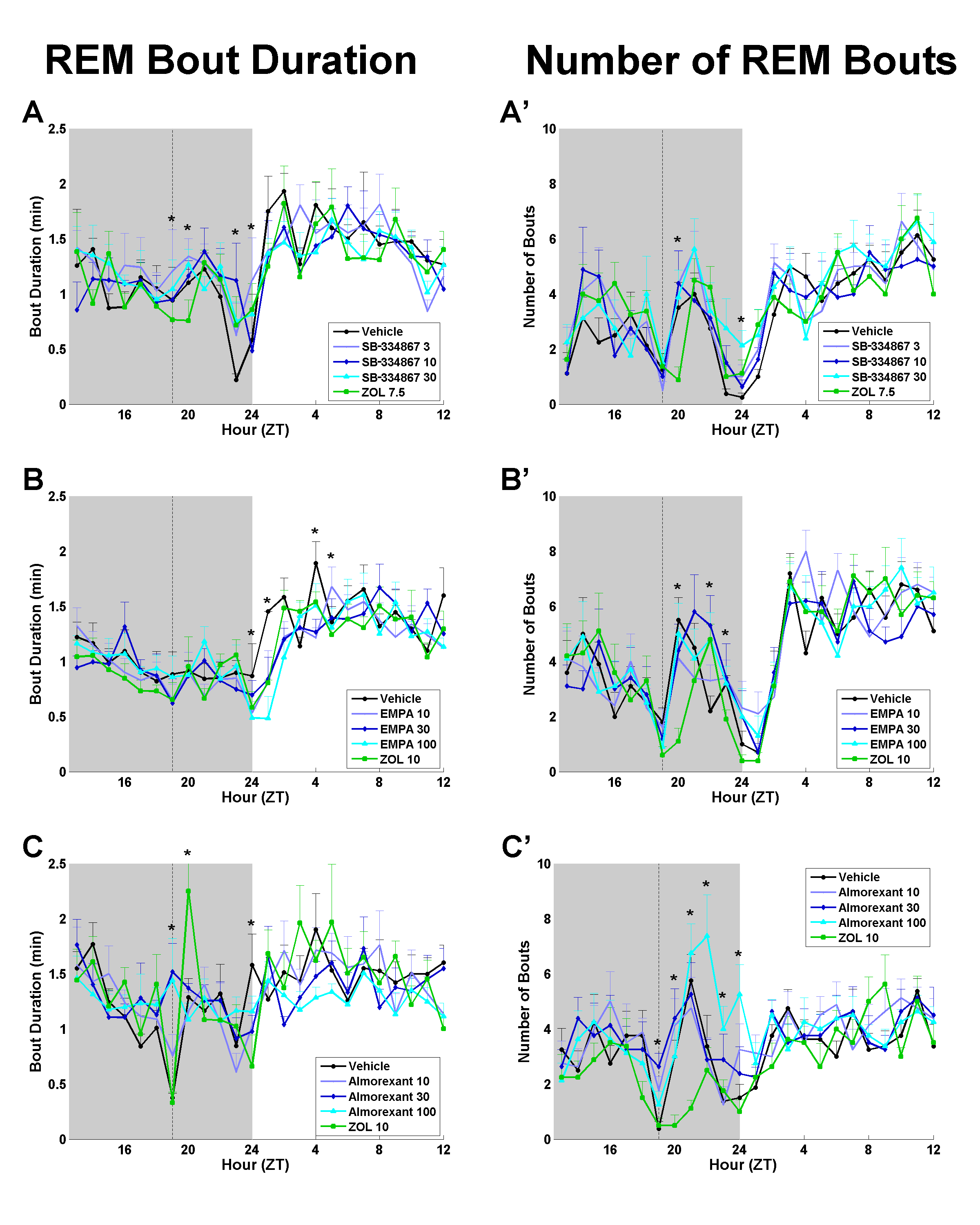

Supplement: Figure S5 — Hourly distribution of REM Sleep Bout Duration and the Number of REM Sleep Bouts. REM Sleep Bout Duration (left) and the Number of REM Sleep Bouts (right) for 6 h prior to and 18 h after administration of SB-334867 (A), EMPA (B), and almorexant (C) as compared to zolpidem (ZOL). Shaded area represents the dark phase; vertical dotted line shows the first h following injection. A: The REM Bout Duration for 3 concentrations of SB 334867 vs. ZOL and vehicle. ANOVA for ZT19-ZT24 is significant for treatment (F = 4.40, p = 0.00692) and treatment by time (F = 2.16, p = 0.00500). For treatment by time: ZT19: ZOL < SB 334867 at 3 mg/kg ZT20: ZOL < all other conditions ZT23: vehicle < all other conditions ZT24: SB 334867 at 10mg/kg < ZOL vehicle < SB 334867 at 3 mg/kg A’: The REM Bout Number for 3 concentrations of SB 334867 vs. ZOL and vehicle. ANOVA for ZT19-ZT24 is significant for treatment by time only (F = 4.49, p = 0.00625). For treatment by time: ZT20: ZOL < all other conditions ZT24: vehicle < SB 334867 at 30 mg/kg B: The REM Bout Duration for 3 concentrations of EMPA vs. ZOL and vehicle. ANOVA for ZT19-ZT24 is significant for treatment by time (F = 1.71, p = 0.03515). ANOVA for ZT1-ZT6 is significant for treatment (F = 4.88, p = 0.00015) and for treatment by time (F = 2.81, p = 0.00015). For treatment by time: ZT21: ZOL < EMPA at 100 mg/kg ZT24: EMPA at 100 mg/kg < vehicle ZT1: EMPA at 100 mg/kg < ZOL all other conditions < vehicle ZT4: EMPA at 10 and 30 mg/kg < vehicle ZT5: ZOL < EMPA at 10 mg/kg B’: The REM Bout Number for 3 concentrations of EMPA vs. ZOL and vehicle. ANOVA for ZT19-ZT24 is significant for treatment (F = 3.99, p = 0.00888) and for treatment by time (F = 1.96, p = 0.01112). For treatment by time: ZT20: ZOL < all other conditions ZT22: vehicle < ZOL ZT23: ZOL < vehicle C: The REM Bout Duration for 3 concentrations of Almorexant vs. ZOL and vehicle. ANOVA for ZT19-ZT24 is significant for treatment by time (F = 6.91, p<0.00001). ANOVA for ZT1-ZT6 is si [file pone.0039131.s005.tif]

**Figure S5**

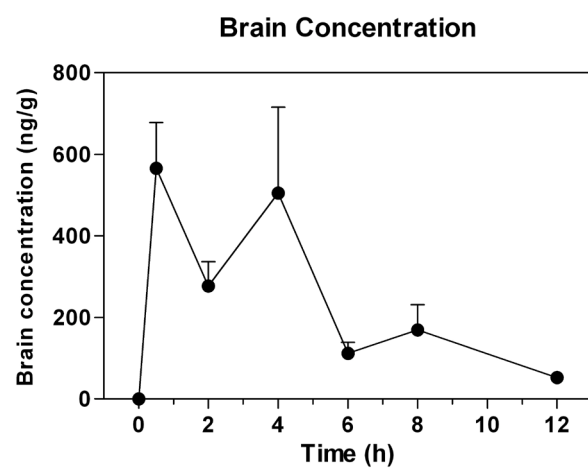

Supplement: Figure S6 — Brain concentration of almorexant. Time course of almorexant concentration in the brain of rats injected intraperitoneally with 30 mg/kg at the mid-dark phase (same animals as in Figures 7). Data are the mean±SEM (n = 5 rats per group). (PDF) [file pone.0039131.s006.pdf]

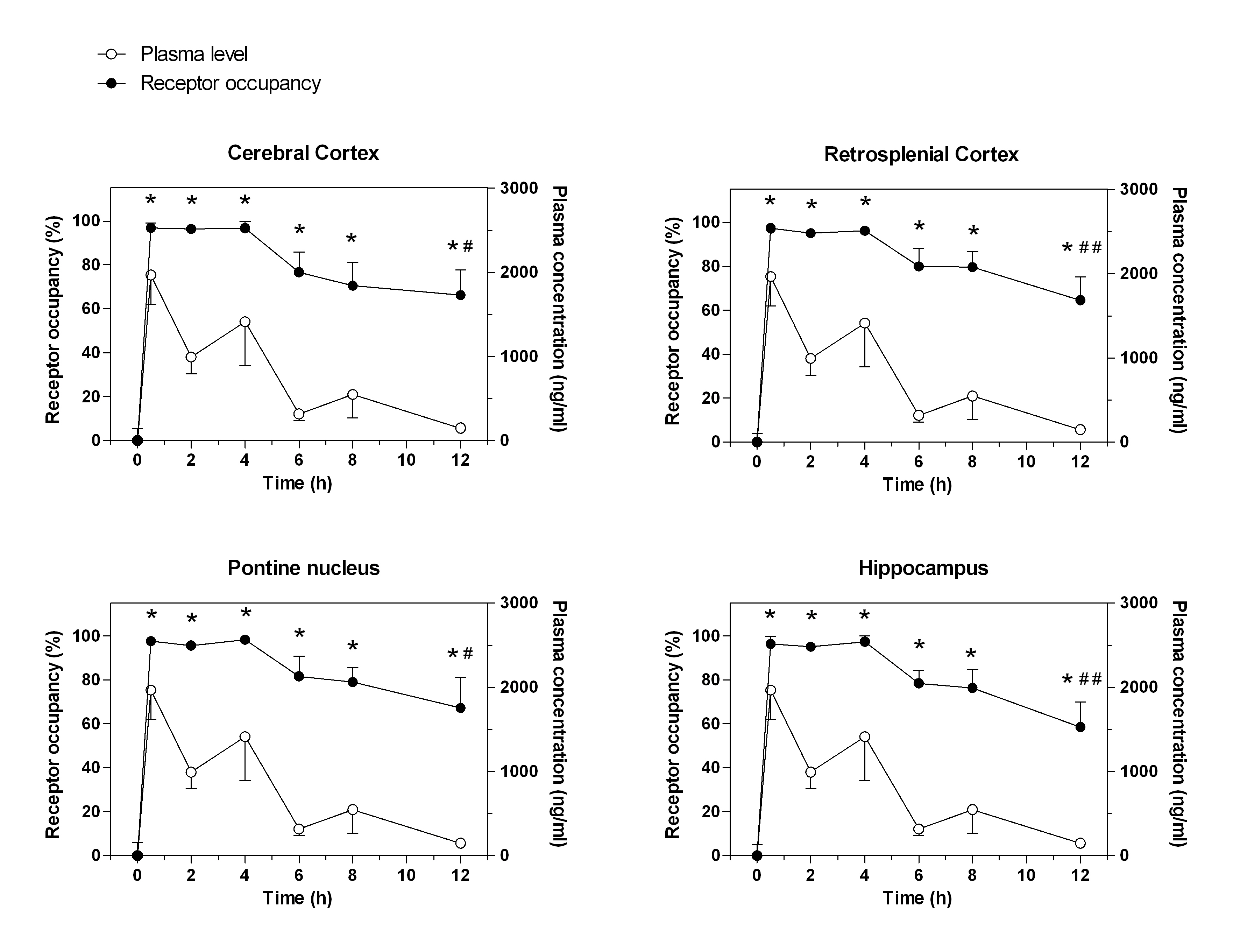

Supplement: Figure S7 — HCRTR2 occupancy in the cerebral cortex, retrosplenial cortex, pontine nuclei, and hippocampus. Data are the mean±SEM (n = 5 rats per group). *, p<0.001 vs. time 0; ##, p<0.01, #, p<0.05 vs. time 30 min (one-way ANOVA followed by Dunnett’s analysis). Almorexant plasma concentrations (data from Figure 7) are shown for comparison. (TIF) [file pone.0039131.s007.tif]
