# Supplementary material for: Dual Hypocretin Receptor Antagonism Is More Effective for Sleep Promotion than Antagonism of Either Receptor Alone
Source: PLoS One. 2012 Jul 2;7(7):e39131. doi: 10.1371/journal.pone.0039131 (PMC3388080; doi:10.1371/journal.pone.0039131)
Supplement: Materials and Methods S1 — Expanded materials and methods for both in vitro and in vivo experiments as referenced in the text. (DOCX) [file pone.0039131.s008.docx]

**Legend with detailed statistics for Figure 5.** Hourly distribution of W, NR and REM sleep for 6 h prior to and 18 h after administration of SB-334867 (**A**), EMPA (**B**), and almorexant (**C**) as compared to zolpidem (ZOL). Shaded area represents the dark phase; vertical dotted line shows the first h following injection.

**A:** The Wake time for 3 concentrations of SB 334867 vs. ZOL and vehicle. ANOVA for ZT19-ZT24 is significant for treatment (F=7.88, p=0.00022). ANOVA for ZT1-ZT6 is significant for treatment (F=2.72, p=0.04989) and for treatment by time (F=1.73, p=0.03501). For treatment by time:

**ZT19:** ZOL < All other conditions

**ZT21:** ZOL < SB 334867 at 10 mg/kg and vehicle

**ZT24:** SB 334867 at 3 and 30 mg/kg and ZOL < vehicle

**ZT3:** SB 334867 at 3 mg/kg and vehicle < ZOL

vehicle < SB 334867 at 10 and 30 mg/kg

**A’:** The NR time for 3 concentrations of SB 334867 vs. ZOL and vehicle. ANOVA for ZT19-ZT24 is significant for treatment (F=10.75, p=0.00002) and for treatment by time (F=1.81, p=0.02486). ANOVA for ZT1-ZT6 is significant for treatment (F=3.57, p=0.01790) and for treatment by time (F=1.66, p=0.04816). For treatment by time:

**ZT19:** all other conditions < ZOL

**ZT21:**  SB 334867 at 10 mg/kg and vehicle < ZOL

vehicle < SB 334867 at 3 mg//kg

**ZT24:** vehicle < SB 334867 at 3 and 30 mg/kg and ZOL

**ZT3:** ZOL < SB 334867 3 mg/kg and vehicle

SB 334867 10 and 30 mg/kg < vehicle

**ZT4:** SB 334867 at 3 mg/kg < vehicle

**A’’:** The REM time for 3 concentrations of SB 334867 vs. ZOL and vehicle. ANOVA for ZT19-ZT24 is significant for treatment (F=5.29, p=0.00267). For treatment by time:

**ZT19:** ZOL < all other conditions

**ZT24:** vehicle < SB 334867 at 30 mg/kg

**B:** The Wake time for 3 concentrations of EMPA vs. ZOL and vehicle. ANOVA for ZT19-ZT24 is significant for treatment (F=7.32, p=0.00020) and for treatment by time (F=2.47, p=0.00087). ANOVA for ZT1-ZT6 is significant for treatment (F=5.88, p=0.00096). For treatment by time:

**ZT19:** ZOL < all other conditions

**ZT20:** ZOL < EMPA at 10 and 30 mg/kg and vehicle

**ZT21:**  ZOL < EMPA at 10 mg/kg

**ZT22:** ZOL < vehicle

**ZT3:** EMPA at 10 mg/kg and vehicle < ZOL

**ZT6:** EMPA at 10 and 30 mg/kg and vehicle < ZOL

**B’:** The NR time for 3 concentrations of EMPA vs. ZOL and vehicle. ANOVA for ZT19-ZT24 is significant for treatment (F=12.82, p<0.00001) and for treatment by time (F=3.20, p=0.00002). ANOVA for ZT1-ZT6 is significant for treatment (F=7.16, p=0.00024). For treatment by time:

**ZT19:** all other conditions < ZOL

**ZT20:** EMPA at 10 and 30 mg/kg and vehicle < ZOL

**ZT21:**  EMPA at 10 mg/kg and vehicle < ZOL

**ZT22:** vehicle < ZOL

**ZT3:** ZOL < EMPA at 10 mg/kg and vehicle

**ZT6:** ZOL < EMPA at 10 and 30 mg/kg and vehicle

**B’’:** The REM time for 3 concentrations of EMPA vs. ZOL and vehicle. ANOVA for ZT19-ZT24 is significant for treatment (F=3.55, p=0.01581) and for treatment by time (F=1.74, p=0.03143). For treatment by time:

**ZT19:** ZOL < vehicle

**ZT20:** ZOL < EMPA at 30 and 100 mg/kg and vehicle

**ZT22:** vehicle < ZOL

**C:** The Wake time for 3 concentrations of Almorexant vs. ZOL and vehicle. ANOVA for ZT19-ZT24 is significant for treatment (F=9.87, p=0.00004) and for treatment by time (F=2.53, p=0.00085). ANOVA for ZT1-ZT6 is significant for treatment (F=4.00, p=0.01093) and for treatment by time (F=1.69, p=0.04140). For treatment by time:

**ZT19:** all other conditions < vehicle

**ZT22:** ZOL < Almorexant at 10 mg/kg

Almorexant at 100 mg/kg < vehicle

**ZT23:** ZOL < Almorexant at 10 mg/kg and vehicle

Almorexant at 100 mg/kg < vehicle

**ZT24:** Almorexant at 100 mg/kg < vehicle

**ZT1:** Almorexant T 30 mg/kg < ZOL and vehicle

**ZT2:** Almorexant at 10 mg/kg and vehicle < ZOL

Vehicle < Almorexant at 100 mg/kg

**ZT6:** Almorexant at 30 mg/kg < ZOL

Almorexant at 30 and 100 mg/kg < vehicle

**C’:** The NR time for 3 concentrations of Almorexant vs. ZOL and vehicle. ANOVA for ZT19-ZT24 is significant for treatment (F=15.18, p<0.00001) and for treatment by time (F=2.42, p=0.00143). ANOVA for ZT1-ZT6 is significant for treatment (F=4.99, p=0.00367) and for treatment by time (F=1.96, p=0.01272). For treatment by time:

**ZT19:** Almorexant at 10 mg/kg and vehicle < ZOL

vehicle < Almorexant at 30 and 100 mg/kg

**ZT20:** Almorexant at 10 mg.kg and vehicle < ZOL

**ZT21:** Almorexant at 10 mg/kg < ZOL

**ZT22:** Almorexant at 10 and 30 mg.kg and vehicle < ZOL

vehicle < Almorexant at 100 mg/kg

**ZT23:** Almorexant at 10 mg/kg and vehicle < ZOL

vehicle < Almorexant at 100 mg/kg

**ZT24:** vehicle < Almorexant at 100 mg/kg

**ZT1:** ZOL and vehicle < Almorexant at 30 mg/kg

**ZT2:** ZOL < Almorexant at 10 and 30 mg/kg and vehicle

**ZT6:** ZOL and vehicle < Almorexant at 30 mg/kg

**C’’:** The REM time for 3 concentrations of Almorexant vs. ZOL and vehicle. ANOVA for ZT19-ZT24 is significant for treatment (F=8.54, p<0.00012) and for treatment by time (F=3.39, p=0.00001). ANOVA for ZT7-ZT12 is significant for treatment by time (F=1.99, p=0.01120). For treatment by time:

**ZT19:** ZOL and vehicle < Almorexant at 30 and 100 mg/kg

**ZT20:** ZOL < Almorexant at 10 and 30 mg/kg

**ZT21:** ZOL < all other conditions

**ZT22:** ZOL < Almorexant at 100 mg/kg

**ZT23:** vehicle < Almorexant at 100 mg/kg

**ZT24:** ZOL < Almorexant at 100 mg/kg and vehicle

vehicle < Almorexant at 100 mg/kg

**ZT8:** Almorexant at 30 mg/kg < ZOL

**ZT9:** Almorexant at 30 and 100 mg/kg and vehicle < ZOL

**ZT10:** ZOL < Almorexant at 10 mg/kg

**Legend with detailed statistics for Figure 6.** Hourly distribution of LMA and T_core_ for 6 h prior to and 18 h after administration of SB-334867 (**A**), EMPA (**B**), and almorexant (**C**) as compared to zolpidem (ZOL). Shaded area represents the dark phase; vertical dotted line shows the first h following injection.

**A:** The average hourly LMA for 3 concentrations of SB-334867 vs. ZOL and vehicle. The dashed line in each panel indicates the first h following dosing. ANOVA for ZT19-ZT24 is significant for treatment (F=4.97, p=0.0037) and for treatment by time (F=1.91, p=0.016). ANOVA for ZT1-ZT6 is significant for treatment by time only (F=2.35, p=0.0020).For treatment by time:

**ZT19:** ZOL < all other conditions.

**ZT20:** ZOL < SB-334867 at 3 mg/kg and vehicle.

**ZT21:** ZOL < SB-334867 at 10 and 30 mg/kg and vehicle.

**ZT24:** ZOL and SB-334867 at 10 and 30 mg/kg < vehicle

**ZT2:** ZOL < SB-334867 at 3 mg/kg.

**ZT3:** Vehicle < SB-334867 at 10 mg/kg and ZOL;

SB-334867 at 3 mg/kg < ZOL.

**ZT6:** SB-334867 at 30 mg/kg< vehicle.

**A’:** The average hourly T_core_ for 3 concentrations of SB-334867 vs. ZOL and vehicle. ANOVA for ZT19-ZT24 is significant for treatment (F=4.92, p=0.0039) and for treatment by time (F=3.60, p<0.0001). ANOVA for ZT1-ZT6 is significant for treatment (F=7.63, p=0.0003) and for treatment by time (F=1.65, p<0.049). ANOVA for ZT7-ZT12 is significant for treatment (F=4.21, p=0.0086). For treatment by time:

**ZT19:** ZOL < SB-334867 at 3 and 10 mg/kg and vehicle.

**ZT20:** SB-334867 at 10 and 30 mg/kg < vehicle;

ZOL < all other conditions.

**ZT23:** SB-334867 at 30 mg/kg and vehicle < ZOL.

**ZT24:** SB-334867 at 30 mg/kg < ZOL.

**ZT1:** SB-334867 at 3 mg/kg < ZOL.

**ZT2:** All other conditions < ZOL.

**ZT3:** All other conditions < ZOL;

Vehicle < SB-334867 at 3 and 30 mg/kg.

**ZT4:** Vehicle < SB-334867 at 30 mg/kg and ZOL;

SB-334867 at 10 mg/kg < ZOL.

**ZT5:** Vehicle < ZOL.

**ZT6:** Vehicle < SB-334867 at 30 mg/kg and ZOL;

SB-334867 at 3 and 10 mg/kg < ZOL..

**ZT8:** SB-334867 at 3 mg/kg and vehicle < ZOL.

**ZT9:** SB-334867 at 3 and 10 mg/kg < ZOL.

**ZT10:** SB-334867 at 3 and 10 mg/kg and vehicle < ZOL.

**ZT11:** Vehicle < ZOL.

**ZT12:** SB-334867 at 3 mg/kg < ZOL.

**B:** The average hourly LMA for 3 concentrations of EMPA vs. ZOL and vehicle. ANOVA for ZT19-ZT24 is significant for treatment (F=7.44, p=0.00018) and for treatment by time (F=3.20, p=0.00002). For treatment by time:

**ZT19:** ZOL < all other conditions.

**ZT20:** ZOL < EMPA at 10 and 30 mg/kg and vehicle;

EMPA at 100 mg/kg < vehicle.

**ZT21:** ZOL < EMPA at 10 mg/kg and vehicle;

EMPA at 30 mg/kg < vehicle.

**ZT22:** ZOL < EMPA at 10 mg/kg and vehicle.

**B’:** The average hourly T_core_ for 3 concentrations of EMPA vs. ZOL and vehicle. ANOVA for ZT19-ZT24 is significant for treatment (F=8.60, P=0.00006) and for treatment by time (F=7.51, P<0.00001). ANOVA for ZT1-ZT6 is significant for treatment (F=15.38, P<0.00001). ANOVA for ZT7-ZT12 is significant for treatment (F=18.22, P<0.00001).For treatment by time:

**ZT19:** ZOL < EMPA at 10 and 30 mg/kg and vehicle;

EMPA at 100 mg/kg < vehicle.

**ZT20:** ZOL < EMPA at 10 and 30 mg/kg and vehicle;

Vehicle < EMPA at 30 mg/kg.

**ZT21:** ZOL < EMPA at 10 and 30 mg/kg and vehicle.

**ZT23:** EMPA at 10 mg/kg and vehicle < ZOL;

Vehicle < EMPA at 100 mg/kg.

**ZT24:** EMPA at 10 and 30 mg/kg and vehicle < ZOL.

**ZT1:** EMPA at 10 and 30 mg/kg < ZOL.

**ZT2:** All other conditions < ZOL.

**ZT3:** All other conditions < ZOL;

Vehicle < SB-334867 at 3 and 30 mg/kg.

**ZT4:** All other conditions < ZOL..

**ZT5:** All other conditions < ZOL;

Vehicle < EMPA at 100 mg/kg.

**ZT6:** All other conditions < ZOL.

**ZT7** All other conditions < ZOL;

Vehicle < EMPA at 100 mg/kg.

**ZT8:** All other conditions < ZOL;

Vehicle < EMPA at 100 mg/kg.

**ZT9:** All other conditions < ZOL.

**ZT10:** All other conditions < ZOL;

Vehicle < EMPA at 100 mg/kg.

**ZT11:** All other conditions < ZOL;

Vehicle < EMPA at 100 mg/kg.

**ZT12:** All other conditions < ZOL;

Vehicle < EMPA at 100 mg/kg.

**C:** The average hourly LMA for 3 concentrations of almorexant vs. ZOL and vehicle. ANOVA for ZT19-ZT24 is significant for treatment (F=7.31, p=0.00036) and for treatment by time (F=2.38, p=0.0018). For treatment by time:

**ZT19:** ZOL < all other conditions.

**ZT20:** ZOL < almorexant at 10 mg/kg and vehicle.

**ZT21:** ZOL < almorexant at 10 and 30 mg/kg and vehicle.

**ZT22:** ZOL < almorexant at 30 mg/kg and vehicle.

**ZT23:** ZOL < almorexant at 10 and 30 mg/kg and vehicle.

**ZT24:** Almorexant at 100 mg/kg and ZOL < vehicle.

**C’:** The average hourly T_core_ for 3 concentrations of almorexant vs. ZOL and vehicle. ANOVA for ZT19-ZT24 is significant for treatment (F=8.60, p=0.00006) and for treatment by time (F=7.51, p<0.00001). ANOVA for ZT1-ZT6 is significant for treatment (F=15.38, p<0.00001) and for treatment by time (F=1.95, p=0.0134). ANOVA for ZT7-ZT12 is significant for treatment (F=7.92, p=0.00021) and for treatment by time (F=1.90, p=0.0167). For treatment by time:

**ZT19:** Almorexant at 100 mg/kg < vehicle.

**ZT20:** ZOL < almorexant at 30 mg/kg and vehicle.

**ZT21:** ZOL < all other conditions.

**ZT22:** Vehicle < almorexant at 30 mg/kg.

**ZT23:** ZOL < almorexant at 10 mg/kg.

**ZT24:** Almorexant at 10 mg/kg < vehicle.

**ZT1:** Almorexant at all concentrations < ZOL.

**ZT2:** All other conditions < ZOL.

**ZT3:** All other conditions < ZOL.

**ZT4:** All other conditions < ZOL.

**ZT5:** All other conditions < ZOL.

**ZT6:** All other conditions < ZOL.

**ZT7** All other conditions < ZOL.

**ZT8:** All other conditions < ZOL.

**ZT9:** All other conditions < ZOL.

**ZT10:** Almorexant at 10 and 30 mg/kg and vehicle < ZOL.

**ZT11:** Almorexant at 10 mg/kg and vehicle < ZOL;

Vehicle < almorexant at 100 mg/kg.

**ZT12:** Almorexant at 10 and 30 mg/kg and vehicle < ZOL.
