# Supplementary material for: Dual Hypocretin Receptor Antagonism Is More Effective for Sleep Promotion than Antagonism of Either Receptor Alone
Source: PLoS One. 2012 Jul 2;7(7):e39131. doi: 10.1371/journal.pone.0039131 (PMC3388080; doi:10.1371/journal.pone.0039131)
Supplement: Table S1 — Pharmacokinetic assessment of almorexant, SB-334867 and SB-408124 after i.v. and p.o. administration to Wistar rat. (DOCX) [file pone.0039131.s010.docx]

**Table S1. Pharmacokinetic assessment of almorexant, SB-334867 and SB-408124 after i.v. and p.o. administration to Wistar rat.**

| **Compound** |  | **Almorexant** | | **SB-334867** | | **SB-408124** | |
| --- | --- | --- | --- | --- | --- | --- | --- |
| **Route** |  | **i.v.** | **p.o.** | **i.v.** | **p.o.** | **i.v.** | **p.o.** |
| **Dose** | mg/kg | 2.40 | 6.35 | 5.18 | 8.75 | 12.10 | 18.16 |
| **C_max_/Dose** | ng/mL | 238.20 | 12.90 | 1937.40 | 331.10 | 4738.30 | 704.8 |
| **T_max_** | h | 0.00 | 0.50 | 0.00 | 1.50 | 0.00 | 2.67 |
| **AUC/Dose** | ng/h/mL | 193.70 | 28.00 | 3118.90 | 1423.50 | 7996.40 | 3455.70 |
| **T_1/2_** | h | 2.58 | 1.29 | 1.21 | 3.10 | 1.12 | 1.95 |
| **Vss** | L/kg | 9.45 |  | 0.57 |  | 0.20 |  |
| **CL** | mL/min/kg | 87.10 |  | 5.40 |  | 2.10 |  |
| **F** | % |  | 14.40 |  | 45.70 |  | 43.20 |
| **Fu** | % |  | <8.70 |  | 0.80 |  | <0.10 |
| **Brain/Plasma** | ratio |  | 0.12 |  | 0.53 |  | 0.03 |

C_max_, maximum concentration; T_max_, time at which maximum concentration was observed; AUC, area under the plasma concentration vs. time curve; CL, clearance; Vss, volume of distribution at steady state; T_1/2_, terminal half-life; F, bioavailability; Fu, Fraction unbound. Plasma values are mean, n=2.
