# Supplementary material for: Dual Hypocretin Receptor Antagonism Is More Effective for Sleep Promotion than Antagonism of Either Receptor Alone
Source: PLoS One. 2012 Jul 2;7(7):e39131. doi: 10.1371/journal.pone.0039131 (PMC3388080; doi:10.1371/journal.pone.0039131)
Supplement: Table S2 — CEREP selectivity screen in the broad radioligand binding assays were undertaken to determine the pharmacological activity of SB-334867. (DOCX) [file pone.0039131.s011.docx]

**Table S2. CEREP selectivity screen in the broad radioligand binding assays were undertaken to determine the pharmacological activity of SB-334867.**

| **Target** | **Reference Compound** | **% Control (10 µM)**  **Mean SB** |
| --- | --- | --- |
|  |  |  |
| **A2A (h)** | **NECA** | **10.6** |
| **A3 (h)** | **IB-MECA** | **37.5** |
| alpha 1 (non-selective) | prazosin | 87.5 |
| alpha 2 (non-selective) | yohimbine | 96.1 |
| beta 1 (h) | atenolol | 85.7 |
| beta 2 (h) | ICI 118551 | 94.6 |
| AT1 (h) | saralasin | 102.3 |
| AT2 (h) | saralasin | 114.4 |
| BZD (central) | diazepam | 89.4 |
| BZD (peripheral) | PK 11195 | 109.3 |
| BB (non-selective) | bombesin | 109.9 |
| B2 (h) | NPC 567 | 97 |
| CGRP (h) | hCGRPalpha | 103.6 |
| CB1 (h) | CP 55940 | 104.1 |
| CCKA (h) (CCK1) | CCK-8 | 108.8 |
| CCKB (h) (CCK2) | CCK-8 | 135.1 |
| D1 (h) | SCH 23390 | 82.7 |
| D2S (h) | (+)butaclamol | 101.2 |
| D3 (h) | (+)butaclamol | 97.1 |
| D4.4 (h) | clozapine | 95.9 |
| D5 (h) | SCH 23390 | 118.6 |
| ETA (h) | endothelin-1 | 97.3 |
| ETB (h) | endothelin-3 | 96.5 |
| GABA (non-selective) | GABA | 105.5 |
| GAL1 (h) | galanin | 118.5 |
| GAL2 (h) | galanin | 97.3 |
| PDGF | PDGF BB | 102.6 |
| CXCR2 (h) (IL-8B) | IL-8 | 110.5 |
| TNF-alpha (h) | TNF-alpha | 96.8 |
| CCR1 (h) | MIP-1alpha | 94.5 |
| H1 (h) | pyrilamine | 100.3 |
| H2 (h) | cimetidine | 86.6 |
| MC4 (h) | NDP-alpha -MSH | 97.9 |
| MT1 (h) | melatonin | 102.4 |
| **ML2 (MT3)** | **melatonin** | **-1.6** |
| MT2 (h) (agonist site) | melatonin | 98.6 |
| M1 (h) | pirenzepine | 103.6 |
| M2 (h) | methoctramine | 87.3 |
| M3 (h) | 4-DAMP | 73.1 |
| M5 (h) | 4-DAMP | 103.3 |
| NK1 (h) | [Sar9,Met(O2)11]-SP | 104.4 |
| NK2 (h) | [Nle10]-NKA(4-10) | 96 |
| NK3 (h) | SB 222200 | 86.3 |
| Y1 (h) | NPY | 102.7 |
| Y2 (h) | NPY | 113 |
| NT1 (h) (NTS1) | neurotensin | 106.9 |
| delta 2 (h) (DOP) | DPDPE | 86.1 |
| kappa (KOP) (guinea-pig) | U 50488 | 100.8 |
| mu (h) (MOP) (agonist site) | DAMGO | 86.3 |
| ORL1 (h) (NOP) | nociceptin | 102.9 |
| PACAP (h) (PAC1) | PACAP1-38 | 107.7 |
| PCP | MK 801 | 91.8 |
| TXA2/PGH2 (h) (TP) | U 44069 | 98.8 |
| P2X | alpha ,beta -MeATP | 94.9 |
| **P2Y** | **dATPalpha S** | **36.4** |
| 5-HT1A (h) | 8-OH-DPAT | 106.6 |
| 5-HT1B | serotonin | 85.9 |
| 5-HT2A (h) | ketanserin | 89.5 |
| **5-HT2C (h)** | **RS-102221** | **30.4** |
| 5-HT3 (h) | MDL 72222 | 93.9 |
| 5-HT5A (h) | serotonin | 84.1 |
| 5-HT6 (h) | serotonin | 102.9 |
| 5-HT7 (h) | serotonin | 99.9 |
| sigma (non-selective) | haloperidol | 98.6 |
| sst (non-selective) | somatostatin-14 | 88.9 |
| TRH1 (h) | TRH | 118.5 |
| VIP1 (h) (VPAC1) | VIP | 101 |
| V1a (h) | [d(CH2)51,Tyr(Me)2]-AVP | 106.1 |
| Ca2+ channel (L, verapamil site) (phenylalkylamines) | D 600 | 92.3 |
| K+V channel | alpha -dendrotoxin | 108.1 |
| SK+Ca channel | apamin | 114.4 |
| Na+ channel (site 2) | veratridine | 62.8 |
| Cl- channel | picrotoxinin | 90.6 |
| NE transporter (h) | protriptyline | 49.4 |
| DA transporter (h) | BTCP | 72.4 |
| 5-HT transporter (h) | imipramine | 110.7 |
| TRH1 (h) (antagonist effect) | chlordiazepoxide | 78.5 |
