# Supplementary material for: Dual Hypocretin Receptor Antagonism Is More Effective for Sleep Promotion than Antagonism of Either Receptor Alone
Source: PLoS One. 2012 Jul 2;7(7):e39131. doi: 10.1371/journal.pone.0039131 (PMC3388080; doi:10.1371/journal.pone.0039131)
Supplement: Table S3 — Measures of state consolidation for 6 h following the administration of SB-334867. (DOCX) [file pone.0039131.s012.docx]

**Table S3.**  **Measures of state consolidation for 6 h following the administration of SB-334867.**

| ZT (hour) | vehicle | SB-334867 | SB-334867 | SB-334867 | ZOL |
| --- | --- | --- | --- | --- | --- |
|  |  | 3 mg/kg | 10 mg/kg | 30 mg/kg | 7.5 mg/kg |
| W bout duration | | | | | |
| 19 | 22.95 ± 8.60 | 31.21 ± 8.79 | 16.83 ± 6.40 | 13.73 ± 6.71 | 6.78 ± 1.81 |
| 20 | 6.52 ± 2.86 | 3.39 ± 0.78 | 9.58 ± 7.23 | 3.30 ± 0.88 | 11.35 ± 6.31 |
| 21 | 3.01 ± 0.39 | 1.44 ± 0.20 | 2.72 ± 0.50 | 2.98 ± 0.68 | 2.44 ± 0.75 |
| 22 | 7.02 ± 3.08 | 6.85 ± 3.36 | 3.15 ± 0.53 | 2.75 ± 0.54 | 3.30 ± 0.66 |
| 23 | 10.99 ± 6.87 | 6.72 ± 1.58 | 11.56 ± 6.94 | 12.52 ± 6.85 | 6.62 ± 0.79 |
| 24 | 29.13 ± 9.16 | 14.70 ± 7.18 | 16.01 ± 6.44 | 5.39 ± 6.44 | 12.51 ± 0.82 |
| 6 h Average | 6.05 ± 1.16 | 4.29 ± 0.34 | 4.19 ±0.30 | 3.87 ± 0.21 | 4.50 ± 0.41 |
| Number of W bouts | | | | | |
| 19 | 5.00 ± 1.66 | 2.88 ± 0.93 | 6.25 ± 1.78 | 5.75 ± 1.10 | 6.75 ± 1.31 |
| 20 | 11.38 ± 2.63 | 12.25 ± 2.06 | 13.88 ± 2.94 | 12.63 ± 1.53 | 6.00 ± 0.82 |
| 21 | 13.00 ± 1.54 | 16.38 ± 1.96 | 14.88 ± 1.83 | 13.00 ± 1.90 | 12.00 ± 1.95 |
| 22 | 12.00 ± 2.67 | 13.38 ± 2.85 | 14.13 ± 1.81 | 16.00 ± 2.00 | 14.75 ± 2.49 |
| 23 | 10.63 ± 1.84 | 9.00 ± 1.66 | 8.88 ± 1.59 | 8.38 ± 2.00 | 7.75 ± 1.19 |
| 24 | 3.63 ± 1.34 | 7.00 ± 1.60 | 5.25 ± 1.22 | 8.63 ±1.29 | 7.88 ± 1.63 |
| 6 h Total | 56.25 ± 7.69 | 61.5 ±5.45 | 64.13 ± 3.89 | 65.38 ± 3.54 | 56.0 ± 4.43 |
| NR bout duration | | | | | |
| 19 | 0.87 ± 0.15 | 1.02 ± 0.14^+^ | 1.56 ± 0.59 | 1.29 ± 0.24 | 3.33 ± 0.78* |
| 20 | 1.26 ± 0.22 | 1.29 ± 0.15^+^ | 1.14 ± 0.12^+^ | 1.41 ± 0.26^+^ | 2.95 ± 0.43* |
| 21 | 0.91 ± 0.07 | 1.45 ± 0.28 | 1.13 ± 0.25 | 1.27 ± 0.16* | 1.87 ± 0.20* |
| 22 | 0.90 ± 0.06 | 0.82 ± 0.13 | 0.86 ± 0.10 | 1.19 ± 0.23 | 1.01 ± 0.10 |
| 23 | 0.69 ± 0.06 | 0.77 ± 0.08 | 0.77 ± 0.13 | 0.80 ± 0.14 | 0.82 ± 0.10 |
| 24 | 0.83 ± 0.11 | 1.40 ± 0.24* | 0.99 ± 0.18 | 1.05 ± 0.12 | 0.84 ± 0.09 |
| 6 h Average | 0.93 ± 0.06 | 1.17 ± 0.12 ^+^ | 1.00 ±0.12 ^+^ | 1.18 ± 0.12 ^+^ | 1.63 ± 0.07* |
| Number of NR bouts | | | | | |
| 19 | 6.38 ± 1.90 | 4.63 ± 2.07 | 7.88 ± 2.52 | 7.75 ± 1.93 | 7.50 ± 1.43 |
| 20 | 14.00 ± 3.49 | 16.25 ± 2.43 | 17.50 ± 3.34 | 13.75 ± 1.79 | 7.75 ± 1.29 |
| 21 | 17.63 ± 2.10 | 20.75 ± 2.40 | 18.13 ± 2.03 | 16.75 ± 2.30 | 17.25 ± 2.00 |
| 22 | 11.88 ± 2.21 | 12.75 ± 2.22 | 17.13 ± 2.47 | 13.13 ± 2.17 | 14.88 ± 1.88 |
| 23 | 10.13 ± 1.71 | 10.50 ± 2.01 | 9.88 ± 1.88 | 8.88 ± 2.17 | 7.25 ± 1.06 |
| 24 | 3.00 ± 1.07 | 7.63 ± 2.02 | 5.38 ± 1.45 | 10.88 ± 1.90 | 8.25 ± 1.75 |
| 6 h Total | 63.38 ± 6.84 | 72.88 ± 5.37 | 76.13 ± 4.99 | 71.38 ± 2.20 | 62.88 ± 3.98 |
| REM bout duration | | | | | |
| 19 | 0.94 ± 0.30 | 1.21 ± 0.38^+^ | 0.95 ± 0.09 | 1.04 ± 0.27 | 0.77 ± 0.20 |
| 20 | 1.10 ± 0.12 | 1.34 ± 0.16^+^ | 1.16 ± 0.14^+^ | 1.27 ± 0.14^+^ | 0.76 ± 0.19* |
| 21 | 1.22 ± 0.15 | 1.28 ± 0.18 | 1.38 ± 0.21 | 1.04 ± 0.11 | 1.28 ± 0.11 |
| 22 | 0.98 ± 0.10 | 1.10 ± 0.27 | 1.16 ± 0.25 | 1.25 ± 0.22 | 1.13 ± 0.23 |
| 23 | 0.22 ± 0.06 | 0.63 ± 0.29* | 1.12 ± 0.34* | 0.75 ± 0.17* | 0.72 ± 0.26* |
| 24 | 0.58 ± 0.42 | 1.13 ± 0.39* | 0.48 ± 0.16^+^ | 0.81 ± 0.12 | 0.86 ± 0.24 |
| 6 h Average | 1.09 ± 0.08 | 1.28 ± 0.14^+^ | 1.24 ±0.14^+^ | 1.05 ± 0.08^+^ | 0.99 ± 0.09 |
| Number of REM bouts | | | | | |
| 19 | 1.25 ± 0.59 | 0.50 ± 0.33 | 1.00 ± .050 | 1.50 ± 0.46 | 1.38 ± 0.46 |
| 20 | 3.50 ± 0.93 | 4.38 ± 1.21 | 4.38 ± 1.19 | 3.88 ± 0.99 | 0.88 ± 0.48 |
| 21 | 4.00 ± 0.76 | 5.63 ± 0.65 | 3.75 ± 0.82 | 5.63 ± 1.10 | 4.50 ± 0.78 |
| 22 | 2.75 ± 0.98 | 2.75 ± 1.10 | 3.13 ± 0.67 | 3.38 ± 1.03 | 1.25 ± 0.75 |
| 23 | 0.38 ± 0.18 | 1.00 ± 0.42 | 1.50 ± 0.63 | 2.75 ± 1.08 | 1.00 ± 0.38 |
| 24 | 0.25 ± 0.16 | 1.00 ± 0.42 | 0.63 ± 0.26 | 2.13 ± 0.55 | 1.13 ± 0.48 |
| 6 h Total | 12.25 ± 1.74 | 15.25 ±2.54 | 14.38 ± 1.55 | 19.25 ± 1.50*^+^ | 13.13 ± 1.32 |

ANOVA for W bout duration significant for condition only (F=4.49, p=0.0063).; ANOVA for the number of W bouts N.S.; ANOVA for NR bout duration significant for condition (F=12.46, p<0.0001) and condition by time (F=4.57, p<0.0001); ANOVA for the number of NR bouts N.S.; ANOVA for REM bout duration significant for condition (F=4.40, p=0.0069) and condition by time (F=2.16, p=0.005); ANOVA for the number of REM bouts significant for condition only (F=4.49, p=0.0063); *=significantly different from vehicle (p<0.05), ^+^=significantly different from ZOL (p<0.05).
