# Supplementary material for: Dual Hypocretin Receptor Antagonism Is More Effective for Sleep Promotion than Antagonism of Either Receptor Alone
Source: PLoS One. 2012 Jul 2;7(7):e39131. doi: 10.1371/journal.pone.0039131 (PMC3388080; doi:10.1371/journal.pone.0039131)
Supplement: Table S4 — Measures of state consolidation for 6 h following the administration of EMPA. (DOCX) [file pone.0039131.s013.docx]

**Table S4.** **Measures of state consolidation for 6 h following the administration of EMPA.**

| **ZT (hour)** | **vehicle** | **EMPA** | **EMPA** | **EMPA** | **ZOL** |
| --- | --- | --- | --- | --- | --- |
|  |  | **10 mg/kg** | **30 mg/kg** | **100 mg/kg** | **10 mg/kg** |
| **W bout duration** | | | | | |
| **19** | 15.26± 5.47 | 16.42± 5.00 | 11.89± 5.40 | 13.14± 5.99 | 3.64± 0.51 |
| **20** | 2.95± 0.52 | 9.37± 5.67 | 9.83± 5.62 | 2.46± 0.62 | 3.23± .037 |
| **21** | 3.79± 0.90 | 6.55± 2.67 | 3.04± 0.83 | 8.88± 5.82 | 3.10± .059 |
| **22** | 5.82± 1.23 | 7.34± 2.67 | 4.90± 1.31 | 2.84± 0.49 | 2.59± 0.31 |
| **23** | 9.01± 4.92 | 6.10± 1.02 | 11.46± 5.56 | 4.89 ±0.81 | 5.59 ±0.93 |
| **24** | 19.68± 7.10 | 9.17± 2.45 | 16.83± 7.31 | 14.62± 6.09 | 12.90± 3.56 |
| **6 h Average** | 4.88 ± 0.44 | 5.56 ± 0.52 | 5.35 ±0.77 | 3.54 ± 0.20* | 3.94 ± 0.29 |
| **Number of W bouts** | | | | | |
| **19** | 4.80± 1.17 | 4.60± 1.25 | 6.20± 0.98 | 9.80± 2.48 | 8.10± 0.97* |
| **20** | 11.70± 1.01 | 10.70± 1.85 | 8.30± 1.27* | 11.60± 1.11 | 8.60± 1.59 |
| **21** | 12.60± 1.83 | 10.20± 1.61 | 11.70± 1.55 | 12.40± 2.01 | 11.00± 1.74 |
| **22** | 8.70± 1.08 | 8.20± 1.73 | 9.20± 1.32 | 13.20± 1.76 | 12.10± 0.85* |
| **23** | 11.20± 2.83 | 7.90± 1.27 | 8.00± 1.56 | 8.60± 1.01 | 8.90± 1.15 |
| **24** | 4.90± 1.14 | 7.80± 1.76* | 7.10± 1.63* | 8.40± 1.92* | 6.20± 1.31 |
| **6 h Total** | 54.90 ± 4.43 | 50.20 ±5.70 | 51.30 ± 4.95 | 65.10 ± 3.37* | 55.80 ± 3.84 |
| **NR bout duration** | | | | | |
| **19** | 1.62± 0.31 | 1.60± 0.25^+^ | 1.27± 0.15^+^ | 1.11± 0.19^+^ | 3.89± 0.68* |
| **20** | 1.50± 0.33 | 1.15± 0.15^+^ | 1.50± 0.28^+^ | 1.86± 0.28^+^ | 3.13± 0.44* |
| **21** | 1.23± 0.27 | 1.12± 0.17 | 1.52± 0.21 | 1.17± 0.11 | 1.81± 0.27 |
| **22** | 1.38± 0.17 | 1.33± 0.24 | 1.38± 0.15 | 1.32± 0.31 | 1.34± 0.17 |
| **23** | 1.27± 0.18 | 1.39± 0.18 | 0.90± 0.11^+^ | 1.10± 0.20 | 0.98± 0.11 |
| **24** | 1.06± 0.12 | 1.25± 0.18^+^ | 1.45± 0.49 | 1.04± 0.17 | 0.92± 0.20 |
| **6 h Average** | 1.31 ± 0.21 | 1.34 ± 0.17 | 1.43 ±0.14 | 1.28 ± 0.12^+^ | 1.80 ± 0.13 |
| **Number of NR bouts** | | | | | |
| **19** | 8.00 ±1.58 | 7.40 ± 2.00 | 9.90 ± 1.60 | 10.80± 2.39 | 8.90 ± 1.06 |
| **20** | 16.00 ±1.48 | 14.00±2.52 | 12.50± 2.18 | 16.50± 2.03 | 11.60± 1.38 |
| **21** | 15.30± 2.39 | 13.50± 2.15 | 15.90± 2.21 | 17.00± 2.91 | 16.40± 2.10 |
| **22** | 9.80± 1.27 | 12.70± 1.87 | 12.20± 1.65 | 18.40± 2.70 | 16.10± 1.11 |
| **23** | 14.50± 1.95 | 11.00± 1.40 | 10.80± 2.35 | 12.30± 1.25 | 13.00± 1.64 |
| **24** | 7.00± 1.66 | 10.00± 2.22 | 7.50± 1.61 | 9.40± 2.24 | 7.50± 1.58 |
| **6 h Total** | 71.40 ± 5.81 | 68.80 ±6.22 | 68.80 ± 7.18 | 84.80 ± **5**.06* | 73.70 ± 4.34 |
| **REM bout duration** | | | | | |
| **19** | 0.88± 0.20 | 0.64± 0.15 | 0.62± 0.22 | 0.86± 0.19 | 0.65± 0.16 |
| **20** | 0.91± 0.10 | 0.90± 0.18 | 0.87± 0.13 | 0.88± 0.08 | 0.96± 0.27 |
| **21** | 0.84± 0.14 | 0.69 ± 0.11 | 1.01 ± 0.11 | 1.18 ± 0.14^+^ | 0.66± 0.09 |
| **22** | 0.85± 0.15 | 0.84± 0.16 | 0.83± 0.13 | 0.84± 0.17 | 0.97± 0.10 |
| **23** | 0.90± 0.11 | 0.85± 0.09 | 0.75± 0.21 | 0.96± 0.19 | 1.06± 0.14 |
| **24** | 0.87± 0.29 | 0.54± 0.10 | 0.69± 0.13 | 0.49± 0.09* | 0.58± 0.14 |
| **6 h Average** | 0.87 ± 0.08 | 0.79 ± 0.09 | 0.88 ± 0.09 | 0.96 ± 0.09 | 0.88 ± 0.07 |
| **Number of REM bouts** | | | | | |
| **19** | 1.80± 0.51 | 1.60± 0.67 | 1.20± 0.49 | 0.90± 0.46 | 0.60± 0.31 |
| **20** | 5.50± 0.82 | 4.10± 1.11 | 4.40± 0.96^+^ | 5.00± 1.12^+^ | 1.10± 0.48* |
| **21** | 4.50± 0.89 | 3.40± 0.73 | 5.80± 1.35 | 4.10± 1.08 | 3.30± 0.75 |
| **22** | 2.20± 0.55 | 3.30± 0.58 | 5.30± 1.09 | 4.80± 0.98 | 4.80± 0.55* |
| **23** | 3.20± 0.29 | 3.40± 0.65 | 3.40± 1.24 | 3.20± 0.63 | 1.90± 0.35* |
| **24** | 1.00± 0.47 | 2.30± 0.98 | 2.00± 0.94 | 2.00± 1.01 | 0.40± 0.22 |
| **6 h Total** | 18.20 ± 1.44 | 18.10 ± 2.28^+^ | 22.10 ± 3.30^+^ | 20.00 ± **1**.93^+^ | 12.10 ± 1.30* |

ANOVA for W bout duration N.S.; ANOVA for the number of W bouts significant for condition only (F=3.65, p=0.013); ANOVA for NR bout duration significant for condition (F=13.46, p<0.0001) and condition by time (F=5.34, p<0.0001); ANOVA for the number of NR bouts N.S.; ANOVA for REM bout duration significant for condition by time only (F=1.71, p=0.035); ANOVA for the number of REM bouts significant for condition (F=3.99, p=0.0089) and condition by time (F=1.96, p=0.011); *=significantly different from vehicle (p<0.05), ^+^=significantly different from ZOL (p<0.05).
