# Supplementary material for: Dual Hypocretin Receptor Antagonism Is More Effective for Sleep Promotion than Antagonism of Either Receptor Alone
Source: PLoS One. 2012 Jul 2;7(7):e39131. doi: 10.1371/journal.pone.0039131 (PMC3388080; doi:10.1371/journal.pone.0039131)
Supplement: Table S5 — Measures of state consolidation for 6 h following the administration of almorexant. (DOCX) [file pone.0039131.s014.docx]

**Table S5. Measures of state consolidation for 6 h following the administration of almorexant.**

| **ZT (hour)** | **vehicle** | **Almorexant** | **Almorexant** | **Almorexant** | **ZOL** |
| --- | --- | --- | --- | --- | --- |
|  |  | **10 mg/kg** | **30 mg/kg** | **100 mg/kg** | **10 mg/kg** |
| **W bout duration** | | | | | |
| **19** | 17.14 ± 6.30 | 9.90 ± 3.21 | 4.69 ± 1.87 | 3.31 ± 0.65 | 4.15 ± 0.82 |
| **20** | 6.42 ± 3.44 | 2.37 ± 0.70 | 1.96 ± 0.75 | 1.41 ± 0.13^+^ | 2.31 ± 0.33 |
| **21** | 2.09 ± 0.52 | 3.03 ± 0.90 | 1.43 ± 0.30^+^ | 0.93 ± 0.15^+^ | 2.69 ± 0.42 |
| **22** | 2.72 ± 0.33 | 4.74 ± 1.57 | 3.07 ± 0.83 | 1.13 ± 0.15^+^* | 3.13 ± 0.59 |
| **23** | 5.23 ± 1.07 | 12.59 ± 3.87 | 5.53 ± 3.36 | 2.15 ± 0.68 | 3.85 ± 1.44 |
| **24** | 10.56 ± 4.12 | 4.00 ± 1.17 | 4.32 ± 1.06 | 1.92 ± 0.34 | 4.15 ± 0.83 |
| **6 h Average** | 5.26 ± 0.62 | 4.37 ± 1.09 | 2.53 ± 0.41* | 1.59 ± 0.20*^+^ | 2.88 ± 0.16* |
| **Number of W bouts** | | | | | |
| **19** | 4.25 ± 0.90 | 6.63 ± 1.50 | 10.38 ± 1.53* | 12.25 ± 1.74* | 8.75 ± 1.56 |
| **20** | 10.50 ± 2.21 | 14.88 ± 2.05^+^ | 16.38 ± 1.68^+^ | 18.38 ± 1.40*^+^ | 10.00 ± 0.71 |
| **21** | 14.63 ± 1.83 | 13.50 ± 1.73 | 18.38 ± 1.39^+^ | 20.88 ± 2.21^+^ | 10.25 ± 1.56 |
| **22** | 13.38 ± 1.10 | 13.00 ± 2.92 | 14.88 ± 1.99 | 20.00 ± 0.89*^+^ | 10.50 ± 1.21 |
| **23** | 10.38 ± 1.52 | 6.63 ± 1.88 | 15.13 ± 2.71 | 21.00 ± 3.02* | 15.13 ± 3.17 |
| **24** | 7.50 ± 1.86 | 12.00 ± 2.10 | 12.00 ± 2.70 | 15.88 ± 1.14* | 12.63 ± 2.40 |
| **6 h Total** | 61.75 ± 5.60 | 67.50 ± 8.89 | 87.8830 ±8.51* | 109.38 ±7.91*^+^ | 68.00 ± 4.23 |
| **NR bout duration** | | | | | |
| **19** | 0.83 ± 0.20 | 1.27 ± 0.15^+^ | 1.33 ± 0.14^+^ | 1.50 ± 0.14*^+^ | 3.53 ± 0.71* |
| **20** | 1.67 ± 0.20 | 1.31 ± 0.09^+^ | 1.34 ± 0.08^+^ | 1.37 ± 0.12^+^ | 2.90 ± 0.19* |
| **21** | 1.45 ± 0.23 | 1.17 ± 0.16^+^ | 1.27 ± 0.09^+^ | 1.31 ± 0.15^+^ | 2.84 ± 0.34* |
| **22** | 1.13 ± 0.11 | 1.07 ± 0.19^+^ | 1.04 ± 0.11^+^ | 1.12 ± 0.15 | 2.27 ± 0.44 |
| **23** | 0.97 ± 0.12 | 1.11 ± 0.19 | 1.03 ± 0.12 | 0.90 ± 0.07 | 1.29 ± 0.20 |
| **24** | 1.56 ± 0.27 | 1.14 ± 0.14 | 1.50 ± 0.24 | 1.14 ± 0.08 | 1.03 ± 0.12 |
| **6 h Average** | 1.31 ± 0.10 | 1.22 ± 0.08^+^ | 1.25 ±0.09^+^ | 1.20 ± 0.08^+^ | 1.99 ± 0.16* |
| **Number of NR bouts** | | | | | |
| **19** | 4.38 ± 1.40 | 8.63 ± 1.82 | 15.00 ± 1.83* | 14.00 ± 1.25* | 9.75 ± 2.02 |
| **20** | 14.25 ± 2.96 | 19.38 ± 2.38 | 19.75 ± 2.25 | 22.25 ± 2.36* | 11.63 ± 1.36 |
| **21** | 18.00 ± 2.60 | 15.88 ± 2.00 | 20.75 ± 1.39 | 23.75 ± 1.47 | 11.88 ± 1.60 |
| **22** | 16.00 ± 0.68 | 14.38 ± 2.58 | 17.25 ± 2.58 | 23.88 ± 1.34* | 13.50 ± 1.88 |
| **23** | 11.88 ± 1.95 | 8.00 ± 1.68 | 16.63 ± 2.88 | 23.13 ± 2.61* | 17.25 ± 3.27 |
| **24** | 9.25 ± 2.27 | 15.25 ± 2.30 | 19.88 ± 2.63 | 19.88 ± 1.14* | 13.13 ± 2.37 |
| **6 h Total** | 73.75 ±7. 55 | 82.00 ±9.10 | 103.0 ± 9.60*^+^ | 127.0 ± 6.02*^+^ | 77.63 ± 3.56 |
| **REM bout duration** | | | | | |
| **19** | 0.38 ± 0.04 | 0.76 ± 0.19*^+^ | 1.52 ± 0.26*^+^ | 1.44 ± 0.38*^+^ | 0.33 ± 0.10 |
| **20** | 1.29 ± 0.17 | 1.16 ± 0.17^+^ | 1.37 ± 0.05^+^ | 1.08 ± 0.15^+^ | 2.25 ± 0.25* |
| **21** | 1.16 ± 0.09 | 1.40 ± 0.16 | 1.26 ± 0.14 | 1.28 ± 0.18 | 1.08 ± 0.19 |
| **22** | 1.32 ± 0.27 | 1.05 ± 0.36 | 1.26 ± 0.17 | 1.07 ± 0.18 | 1.08 ± 0.19 |
| **23** | 0.85 ± 0.16 | 0.61 ± 0.17 | 0.91 ± 0.19 | 1.16 ± 0.12 | 1.02 ± 0.20 |
| **24** | 1.58 ± 0.28 | 1.02 ± 0.21^+^ | 0.98 ± 0.15* | 1.16 ± 0.14^+^ | 0.66 ± 0.29* |
| **6 h Average** | 1.19 ± 0.09 | 1.11 ± 0.13 | 1.32 ± 0.10 | 1.20 ± 0.12 | 0.97 ± 0.12 |
| **Number of REM bouts** | | | | | |
| **19** | 0.38 ± 0.26 | 1.75 ± 0.53 | 2.63 ± 0.50*^+^ | 1.25 ± 0.49 | 0.50 ± 0.27 |
| **20** | 3.00 ± 0.60 | 4.13 ± 0.97^+^ | 4.38 ± 1.08^+^ | 3.00 ± 1.20 | 0.50 ± 0.38* |
| **21** | 5.75 ± 0.96 | 4.75 ± 1.13^+^ | 5.25 ± 1.16^+^ | 6.75 ± 1.06^+^ | 1.13 ± 0.30* |
| **22** | 3.38 ± 1.12 | 2.88 ± 0.81 | 2.88 ± 0.74 | 7.38 ± 1.49*^+^ | 2.50 ± 0.82 |
| **23** | 1.38 ± 0.38 | 1.25 ± 0.65 | 2.88 ± 0.93 | 4.00 ± 0.82* | 1.75 ± 0.41 |
| **24** | 1.50 ± 0.50 | 3.25 ± 0.94 | 2.38 ± 0.98 | 5.25 ± 1.08*^+^ | 1.00 ± 0.42 |
| **6 h Total** | 15.38 ± 2.08 | 18.00 ± 3.80^+^ | 20.38 ± 3.12^+^ | 27.75 ± 3.26*^+^ | 7.38 ± 1.68* |

ANOVA for W bout duration significant for condition (F=4.01 p=0.011) and condition by time (F=2.32, p=0.0023); ANOVA for the number of W bouts significant for condition (F=8.81, p=0.0009) and condition by time (F=2.07, p=0.0077); ANOVA for NR bout duration significant for condition (F=16.44, p<0.0001) and condition by time (F=5.34, p<0.0001); ANOVA for the number of NR bouts significant for condition (F=12.58, p<0.0001) and condition by time (F=2.41, p=0.0015); ANOVA for REM bout duration significant for condition by time only (F=6.91, p<0.0001); ANOVA for the number of REM bouts significant for condition (F=9.29, p<0.0001) and condition by time (F=2.96, p<0.0001); *=significantly different from vehicle (p<0.05), ^+^=significantly different from ZOL (p<0.05).
